# Supplementary material for: Design and Synthesis of New Benzo[d]oxazole-Based Derivatives and Their Neuroprotective Effects on β-Amyloid-Induced PC12 Cells
Source: Molecules. 2020 Nov 18;25(22):5391. doi: 10.3390/molecules25225391 (PMC7698601; doi:10.3390/molecules25225391)
Supplement: Supplementary file 1 [file molecules-25-05391-s001.pdf]

Supporting Information for
Original article

Design and Synthesis of New Benzo[d]oxazole-based Derivatives and Their Neuroprotective Effects on β-Amyloid-Induced PC12 Cells

Zheng Liu [1,#], Ming Bian[1,2,#], Qian-qian Ma[1,2,#], Zhuo Zhang[3], Huan-huan Du[1,2,*] and Cheng-xi Wei [1,2,*]

[1] Medicinal Chemistry and Pharmacology Institute, Inner Mongolia University for the Nationalities, Tongliao City, Inner Mongolia, 028000, P.R. China; 527990796@qq.com (Z.L.); bmz3@163.com (M.B.); maqq2020@126.com (Q.M.); joycetu@126.com (H.D.); weichengxi1224@163.com (C.W.)

[2] Inner Mongolia Key Laboratory of Mongolian Medicine Pharmacology for Cardio-Cerebral Vascular System, Tongliao City, Inner Mongolia, 028000, P.R. China

[3] College of pharmaceutical sciences, Yanbian University, Yanji City, Jilin, 133022, P.R. China; zhangzhuo0523mm@163.com

[#] These authors contributed equally to this work

* Correspondence: joycetu@126.com (H.D.); weichengxi1224@163.com (C.W.); Tel.: 86-475-8314245

1. ^{1}H NMR and ^{13}C NMR spectra of intermediates and target compounds

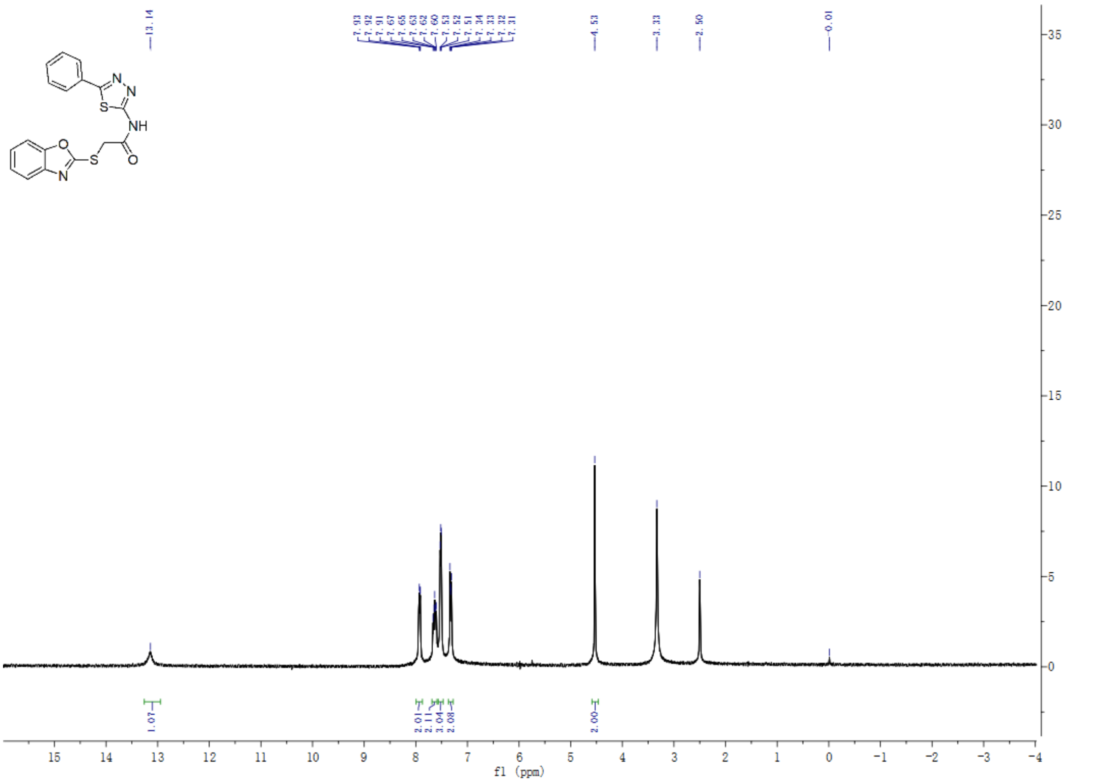

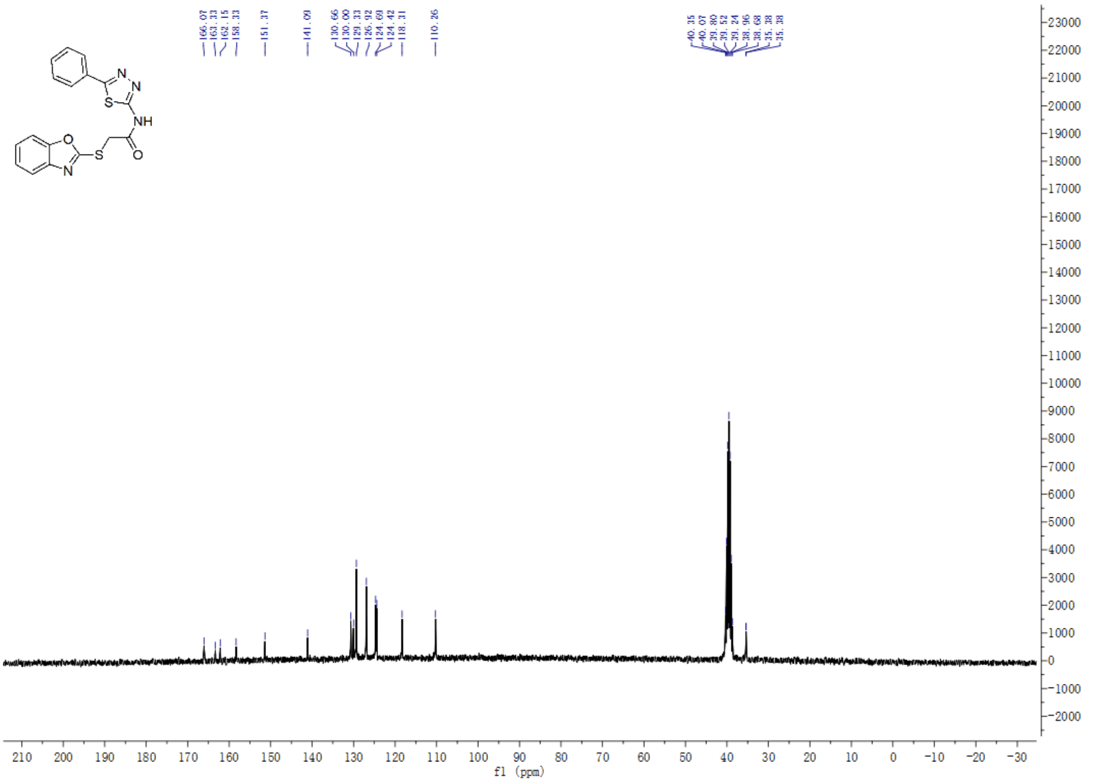

^{1}H NMR and ^{13}C NMR spectra of compound **5a**

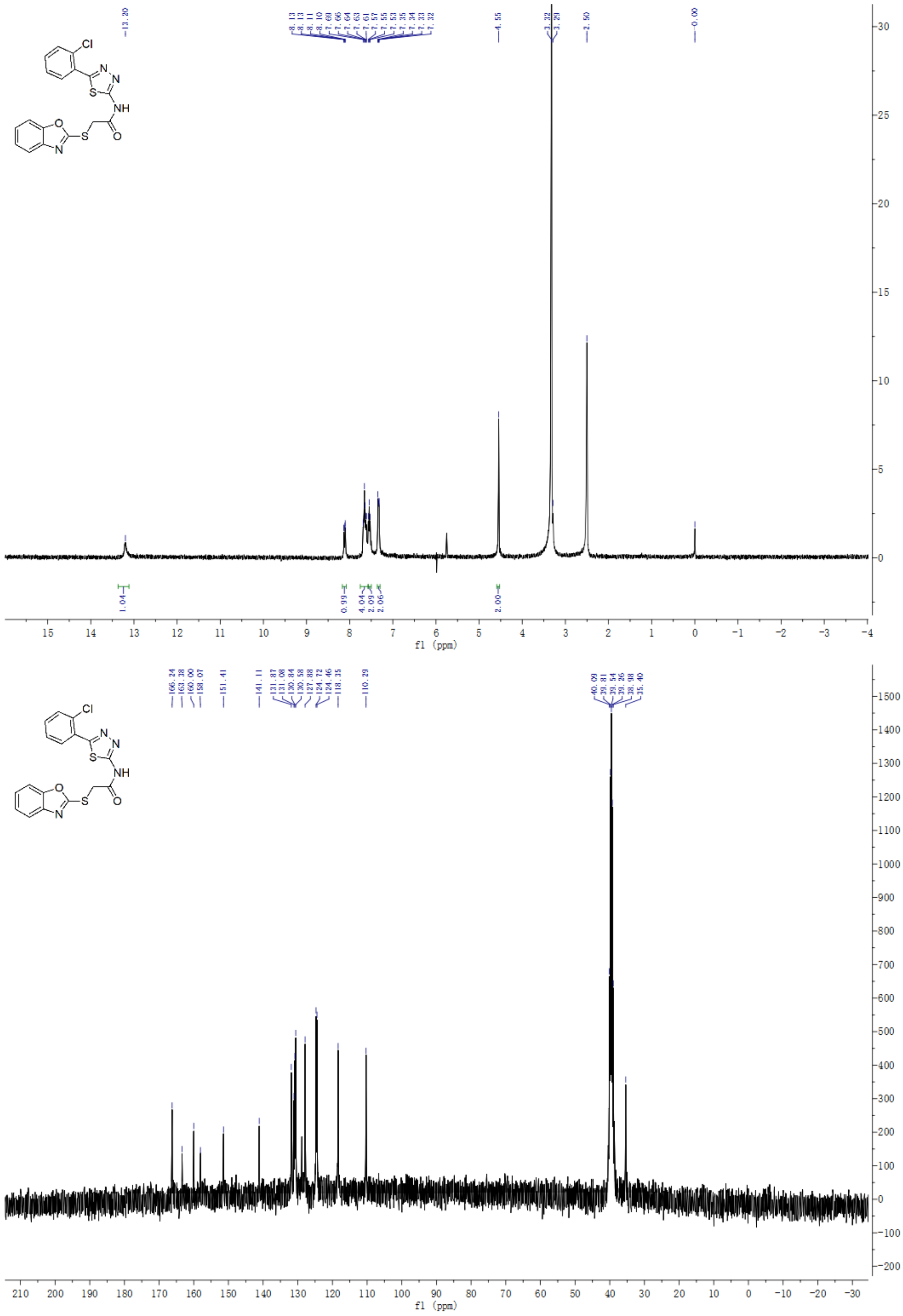

^{1}H NMR and ^{13}C NMR spectra of compound **5b**

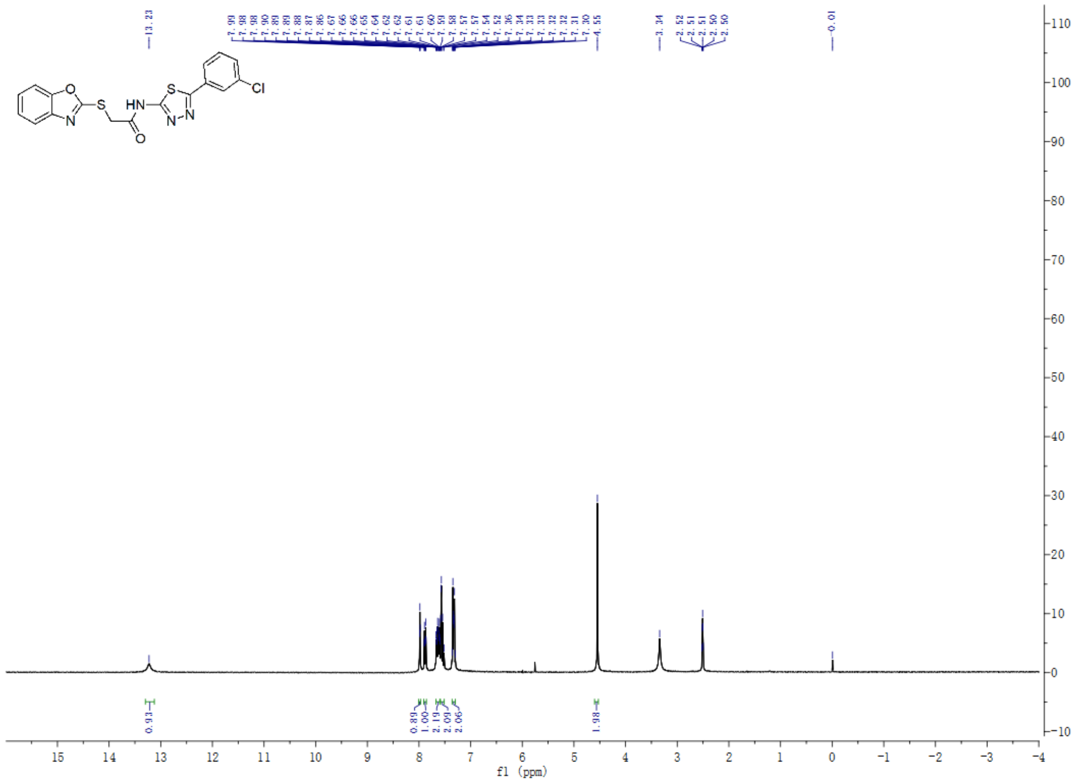

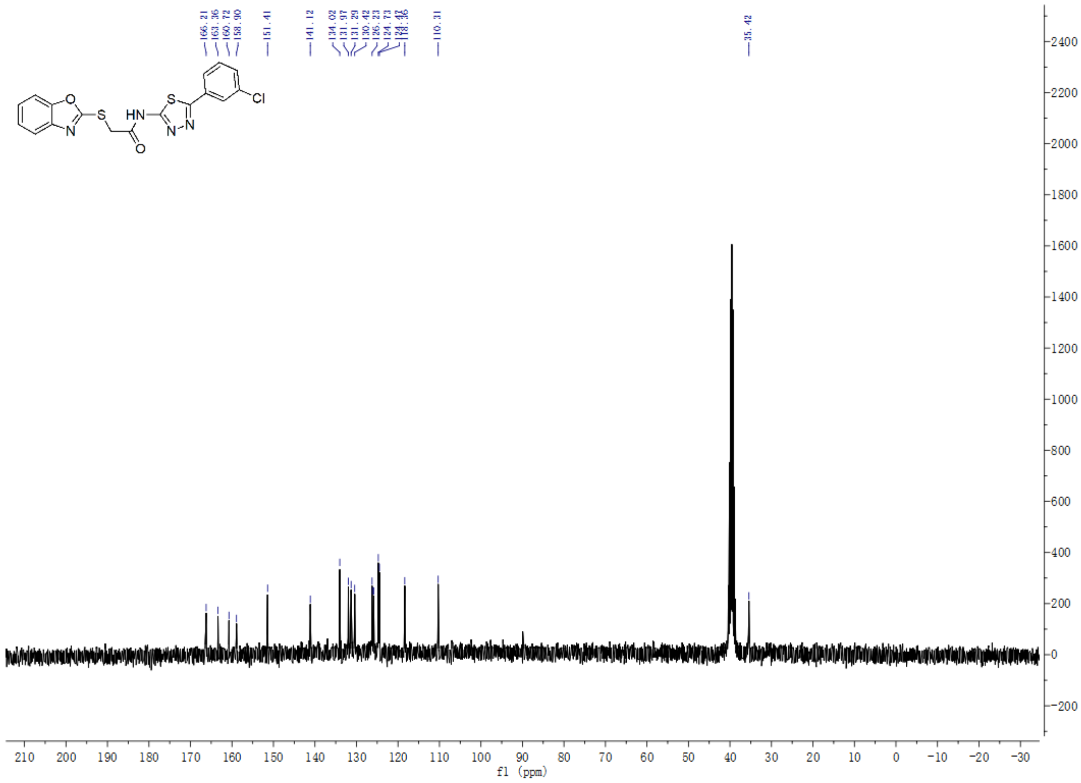

^{1}H NMR and ^{13}C NMR spectra of compound **5c**

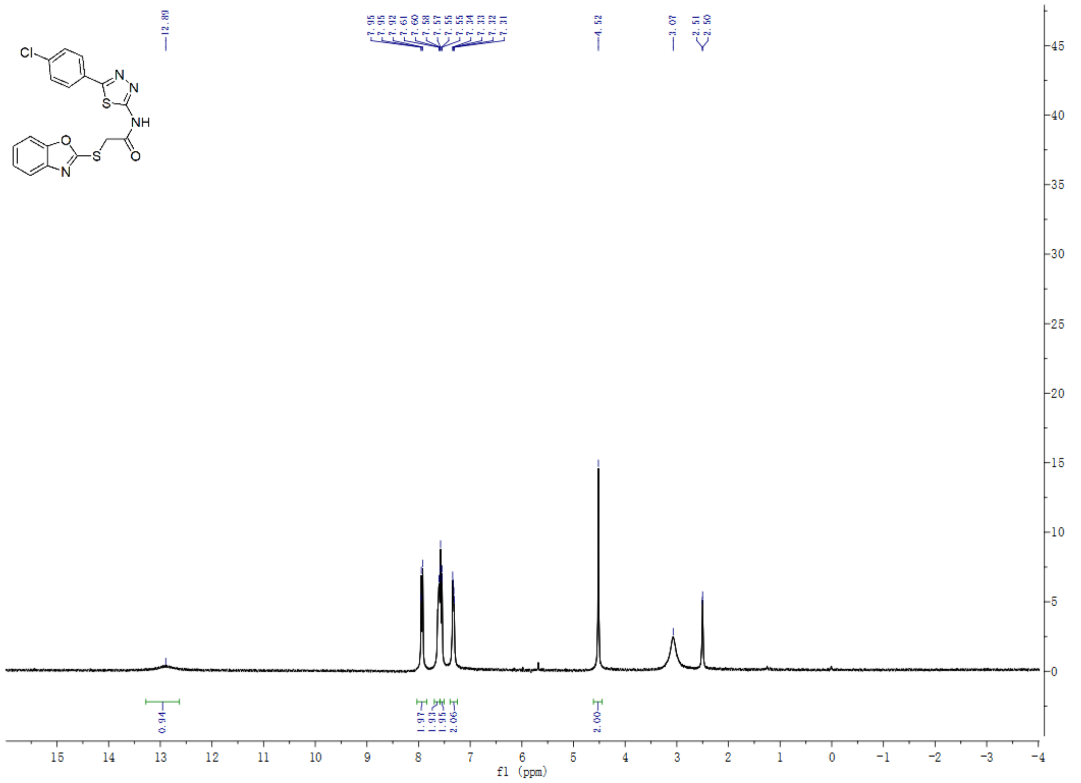

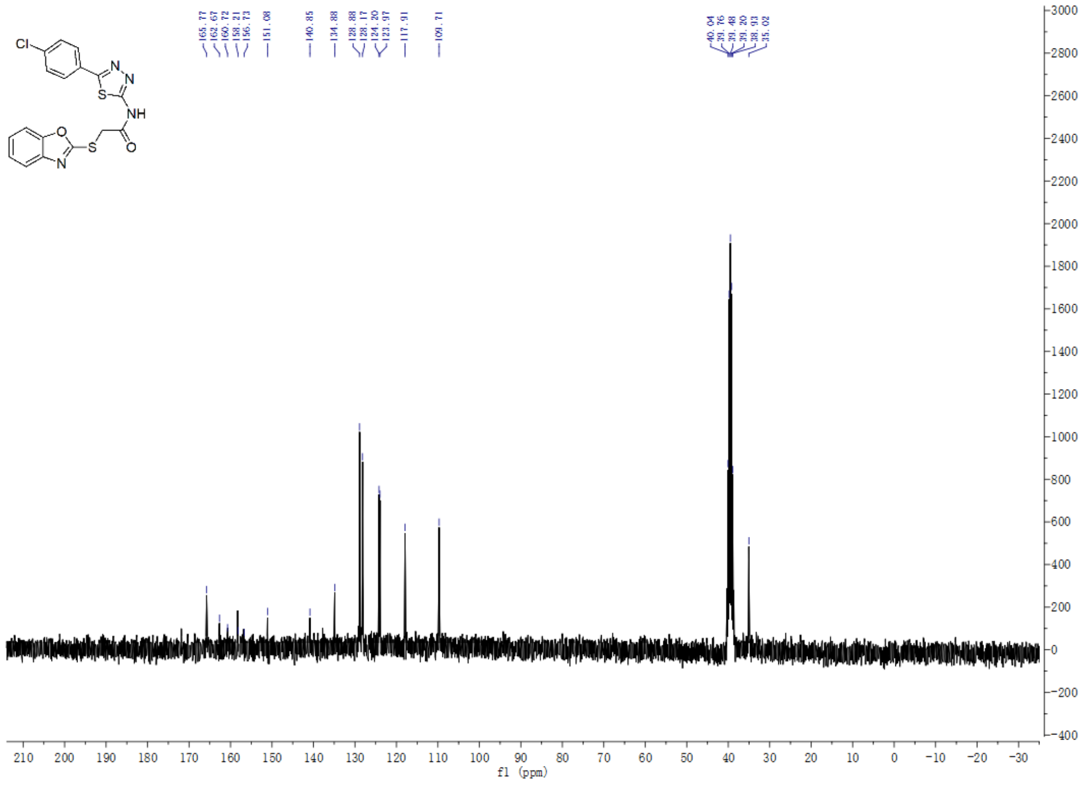

^{1}H NMR and ^{13}C NMR spectra of compound **5d**

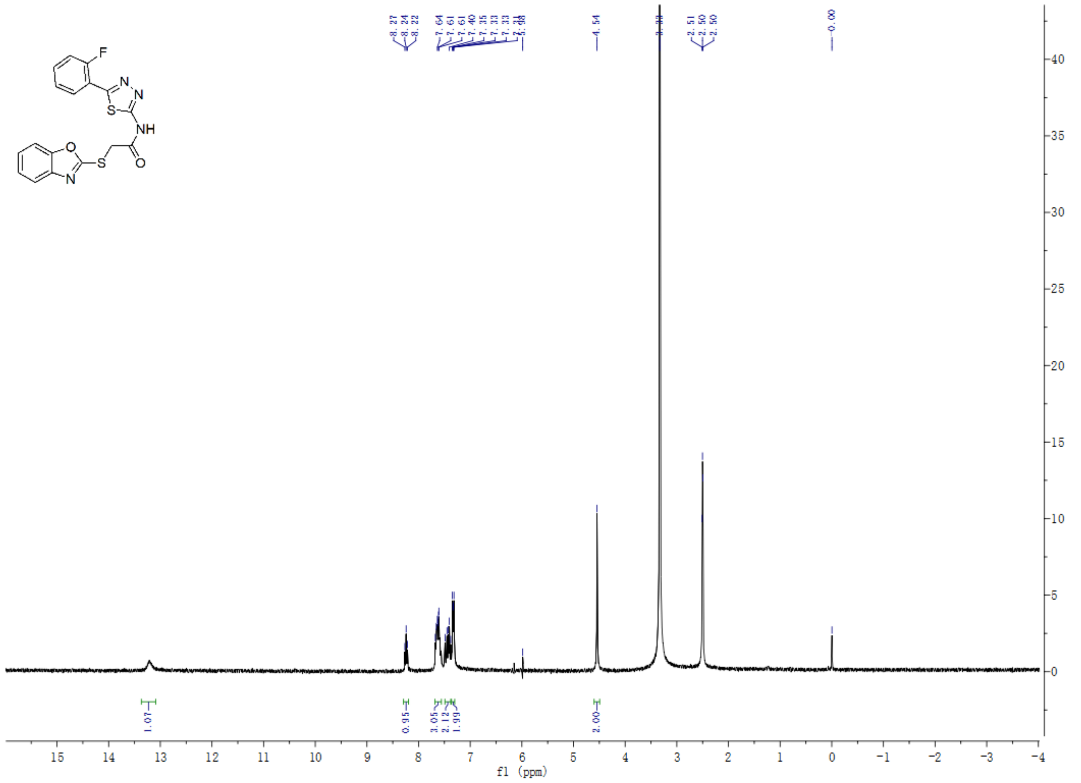

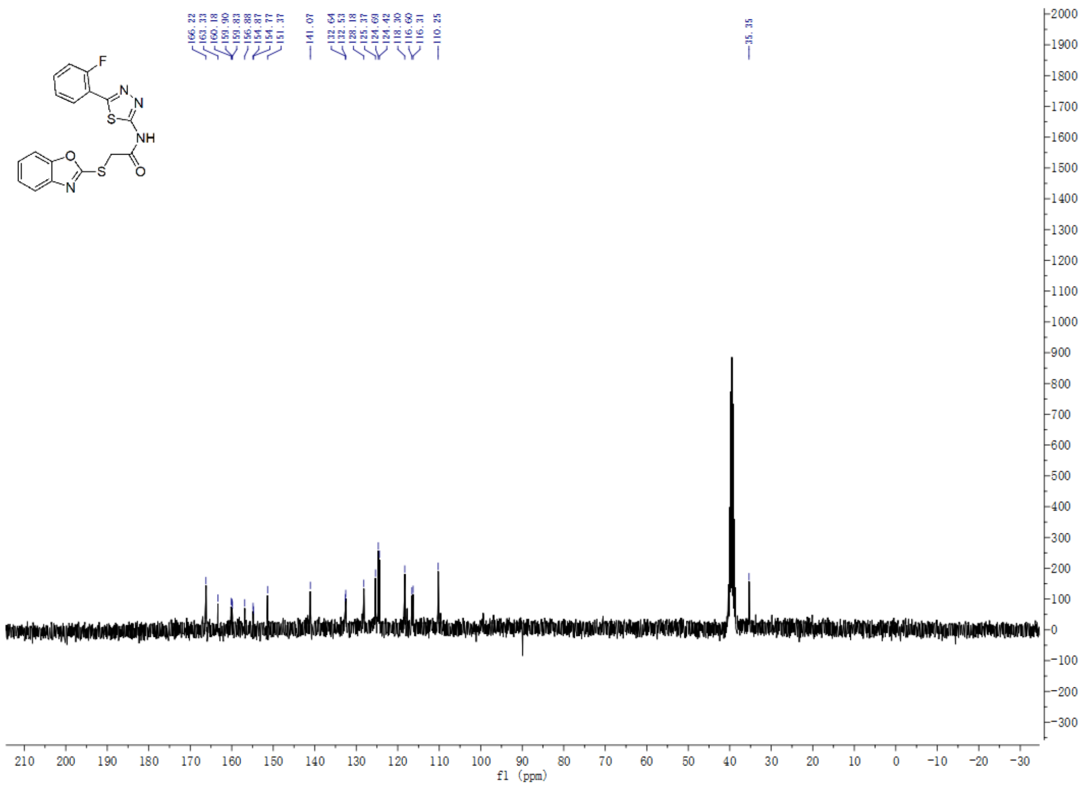

^{1}H NMR and ^{13}C NMR spectra of compound **5e**

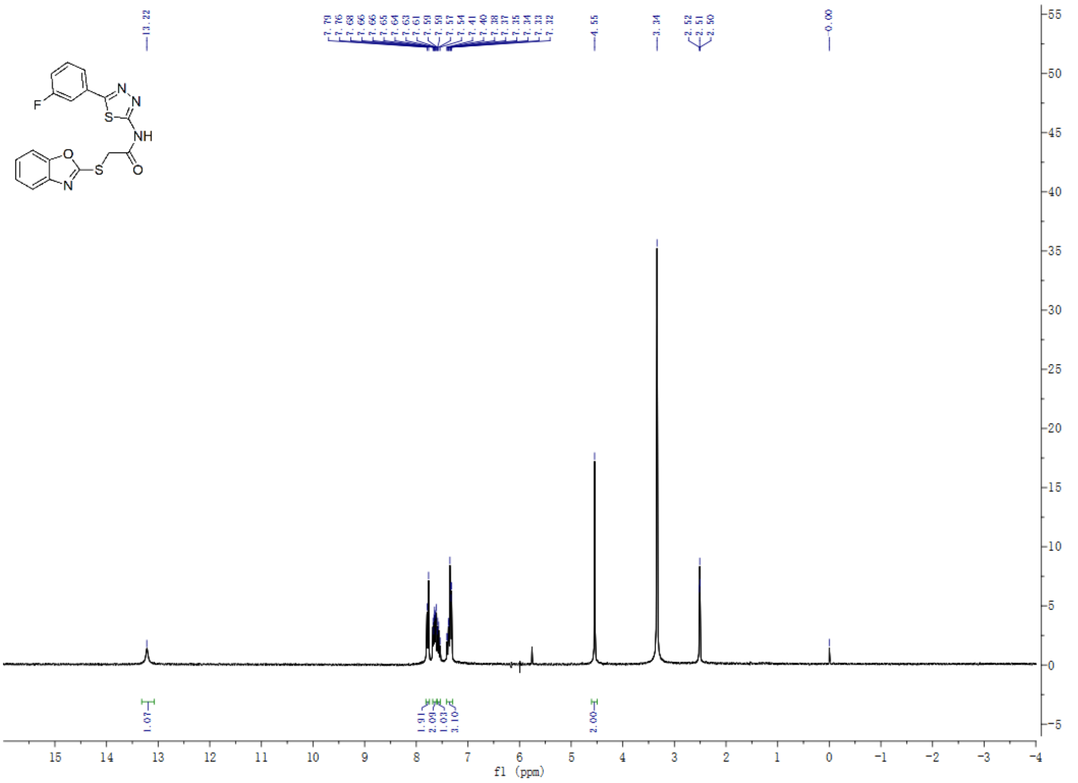

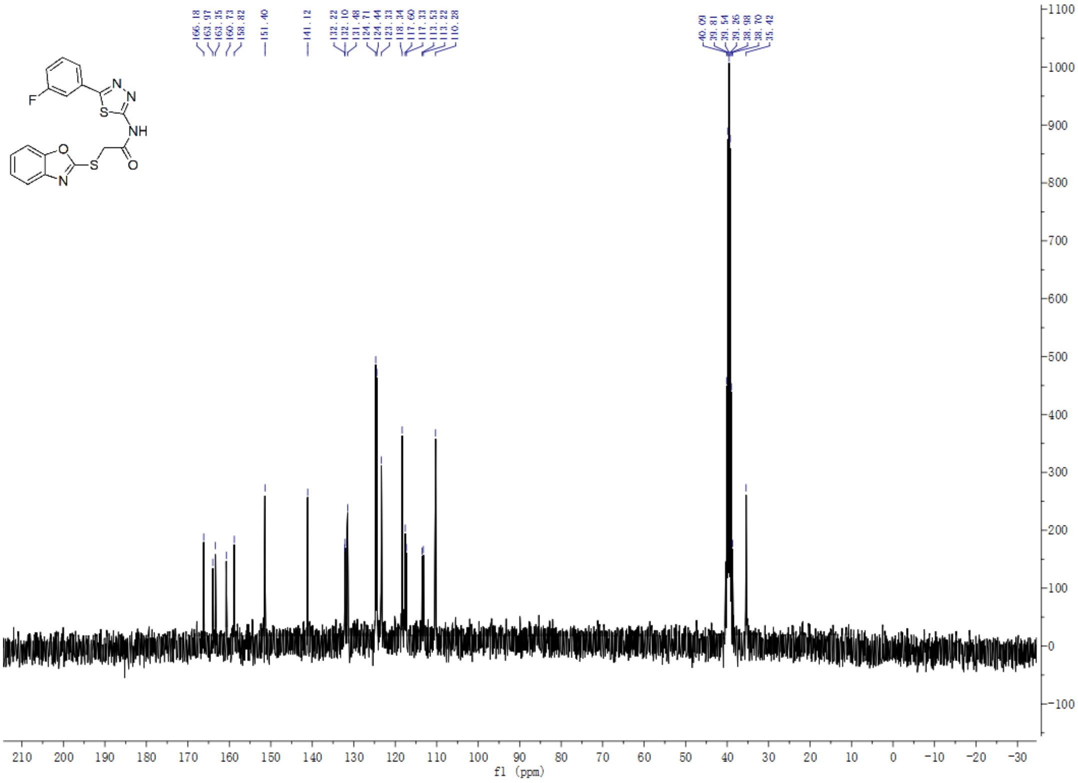

^{1}H NMR and ^{13}C NMR spectra of compound **5f**

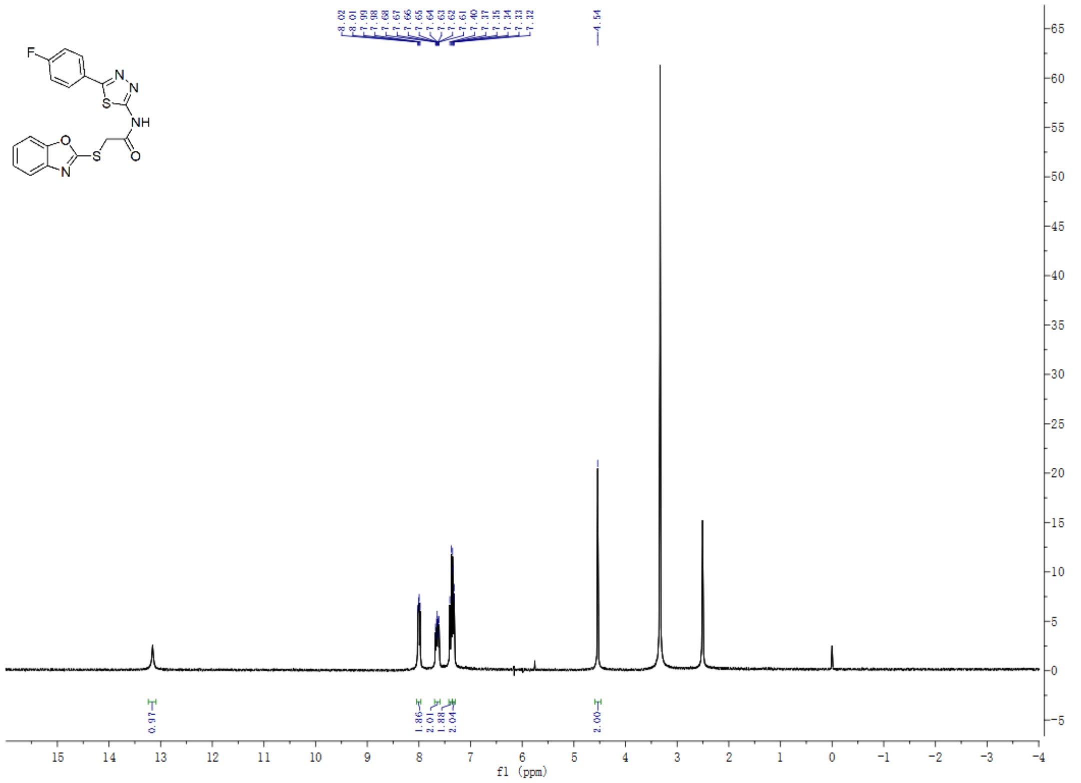

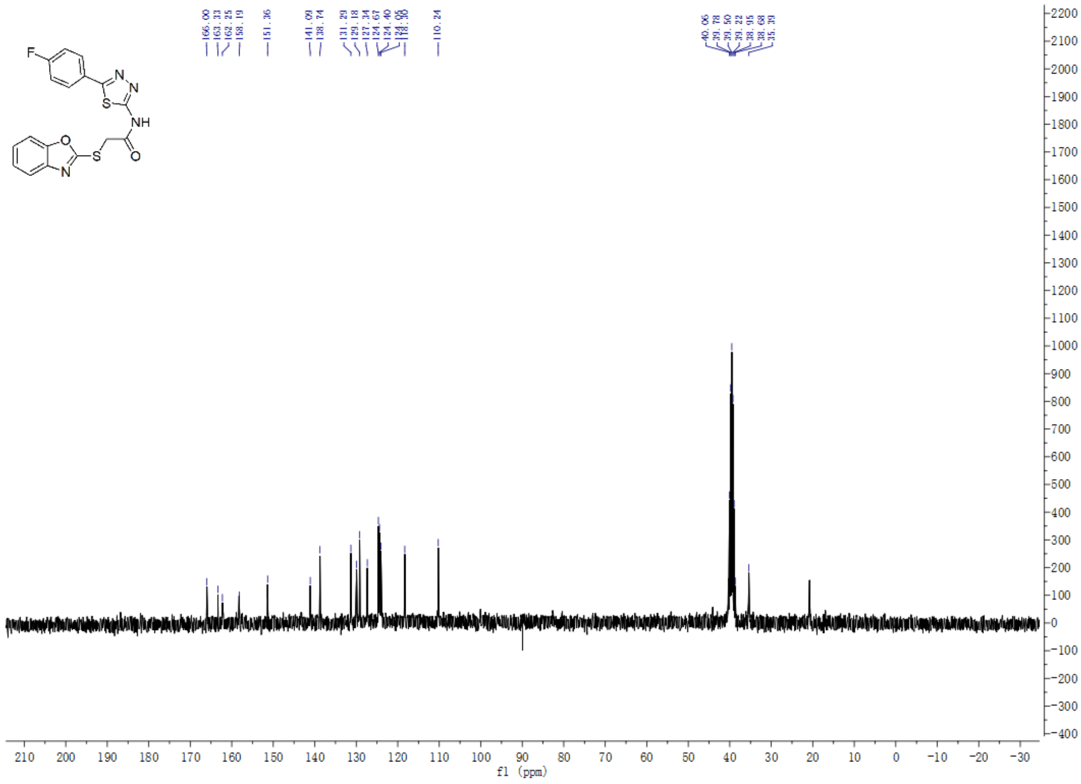

^{1}H NMR and ^{13}C NMR spectra of compound **5g**

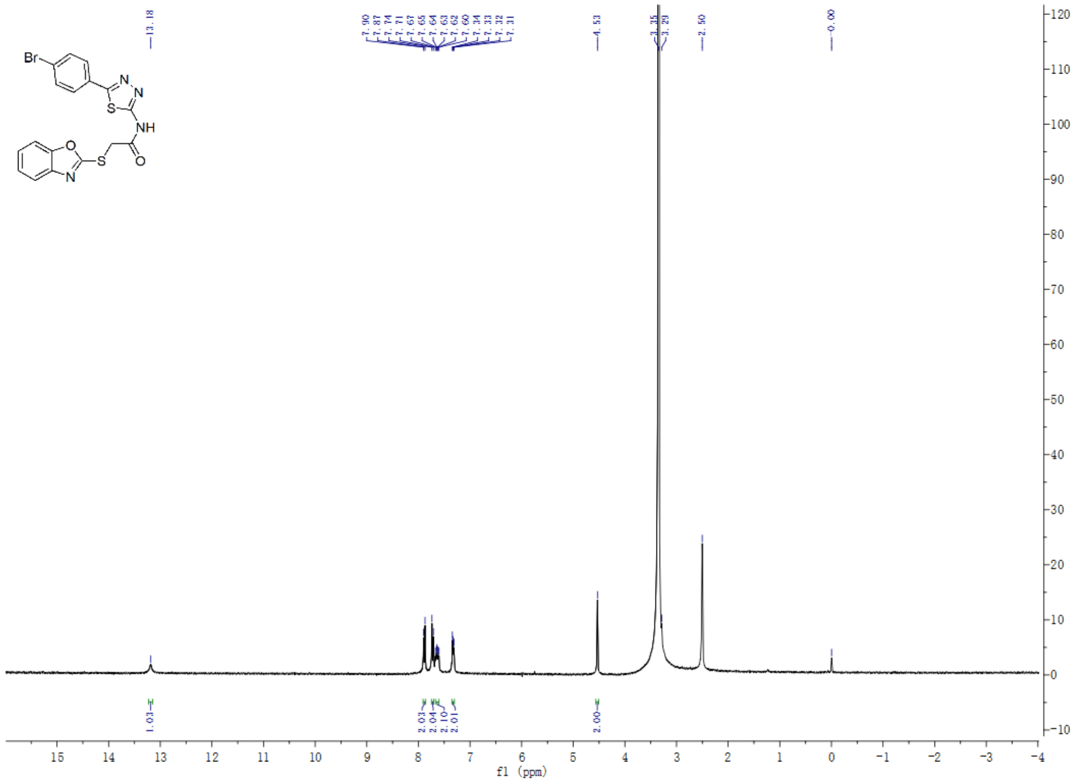

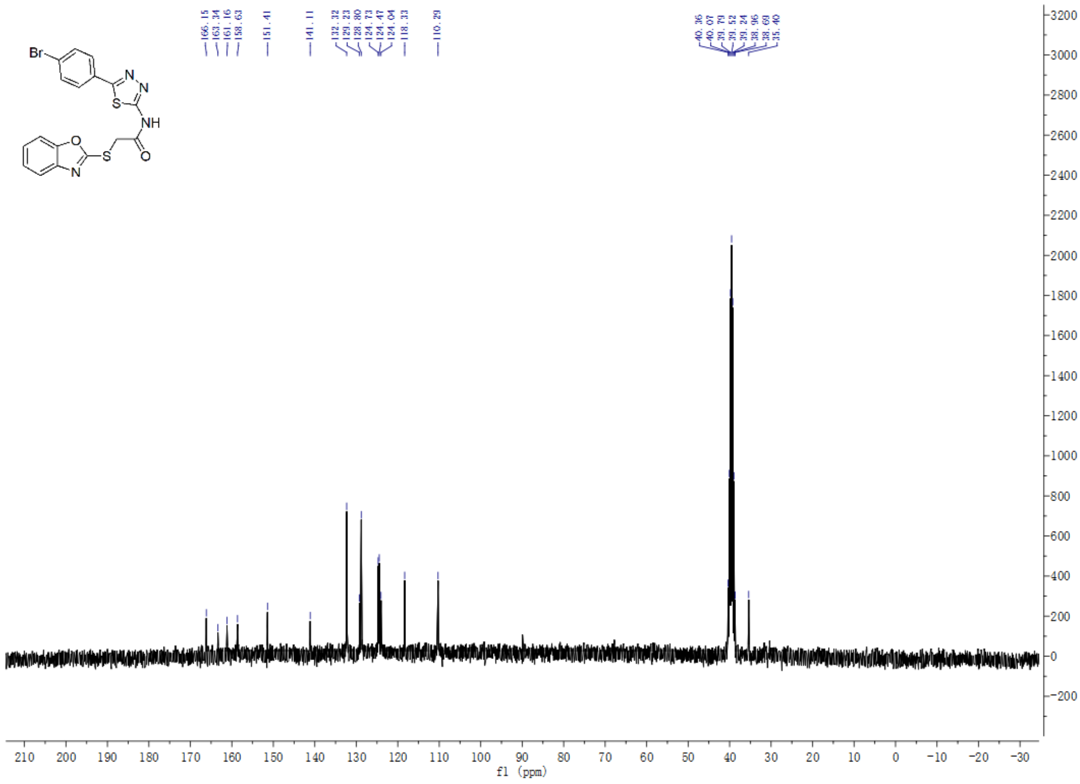

^{1}H NMR and ^{13}C NMR spectra of compound **5h**

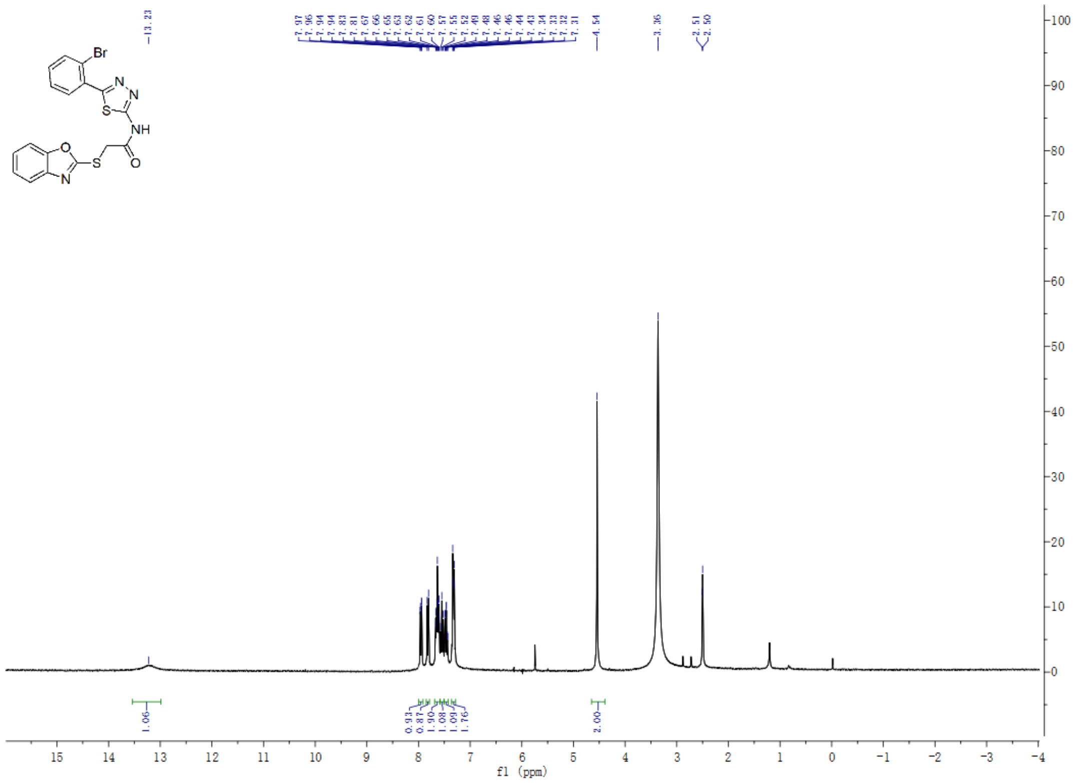

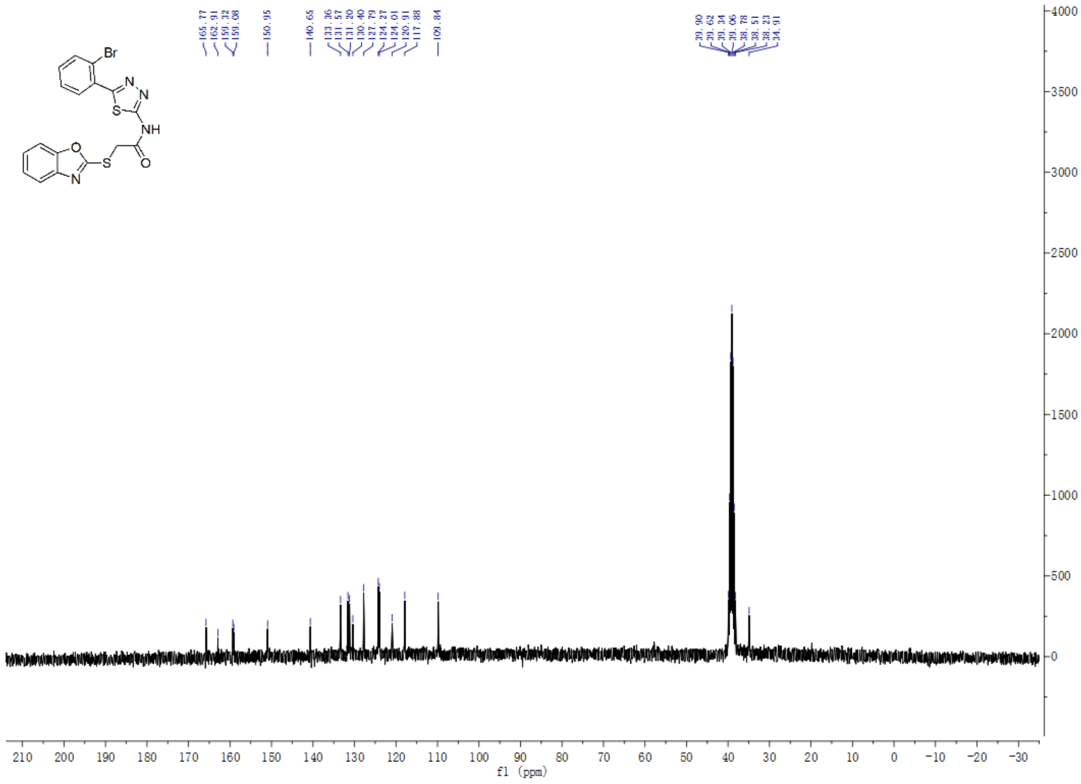

^{1}H NMR and ^{13}C NMR spectra of compound **5i**

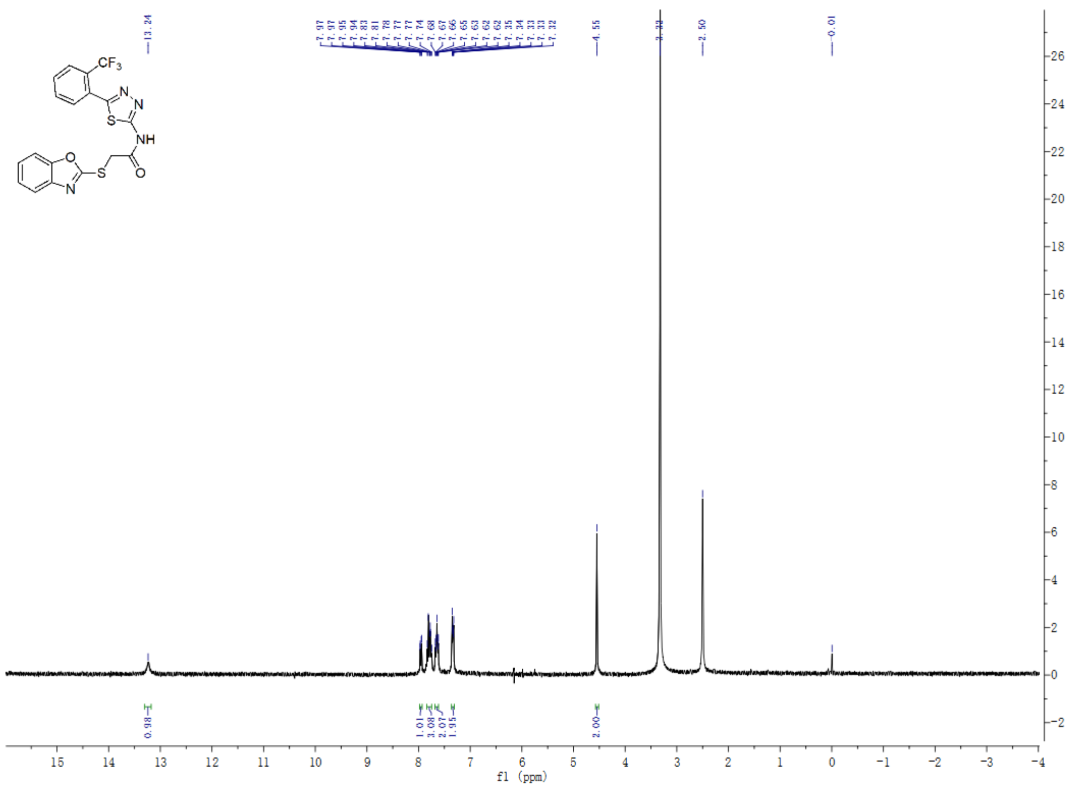

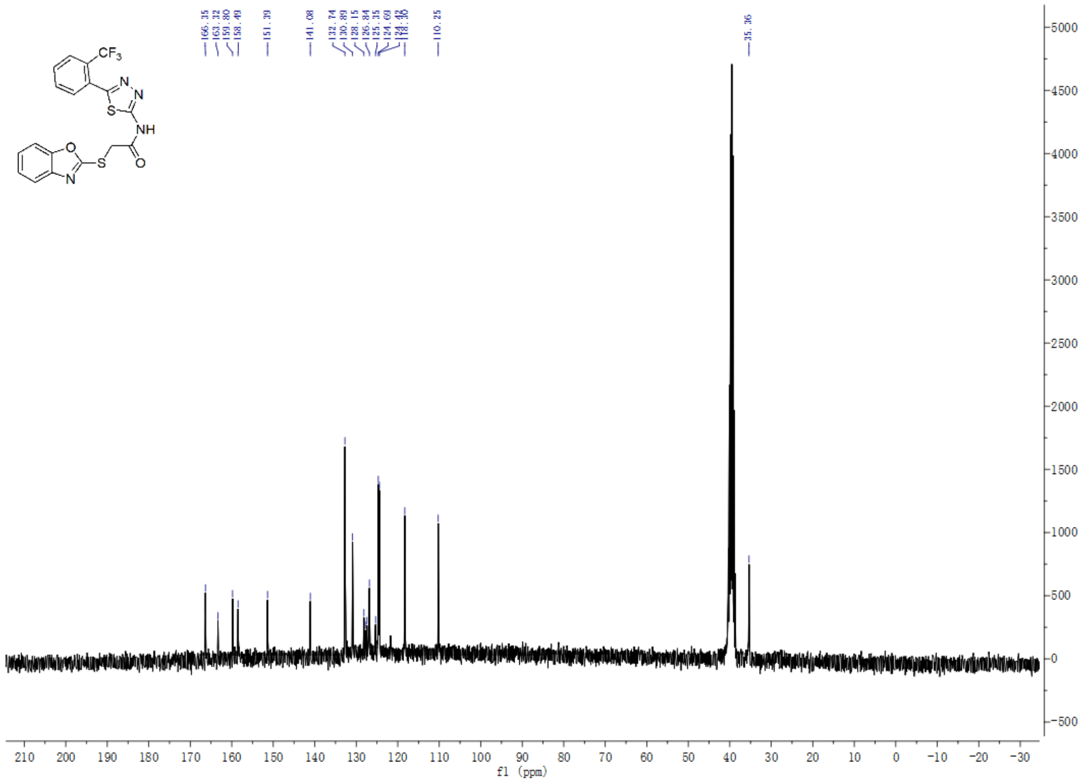

^{1}H NMR and ^{13}C NMR spectra of compound **5j**

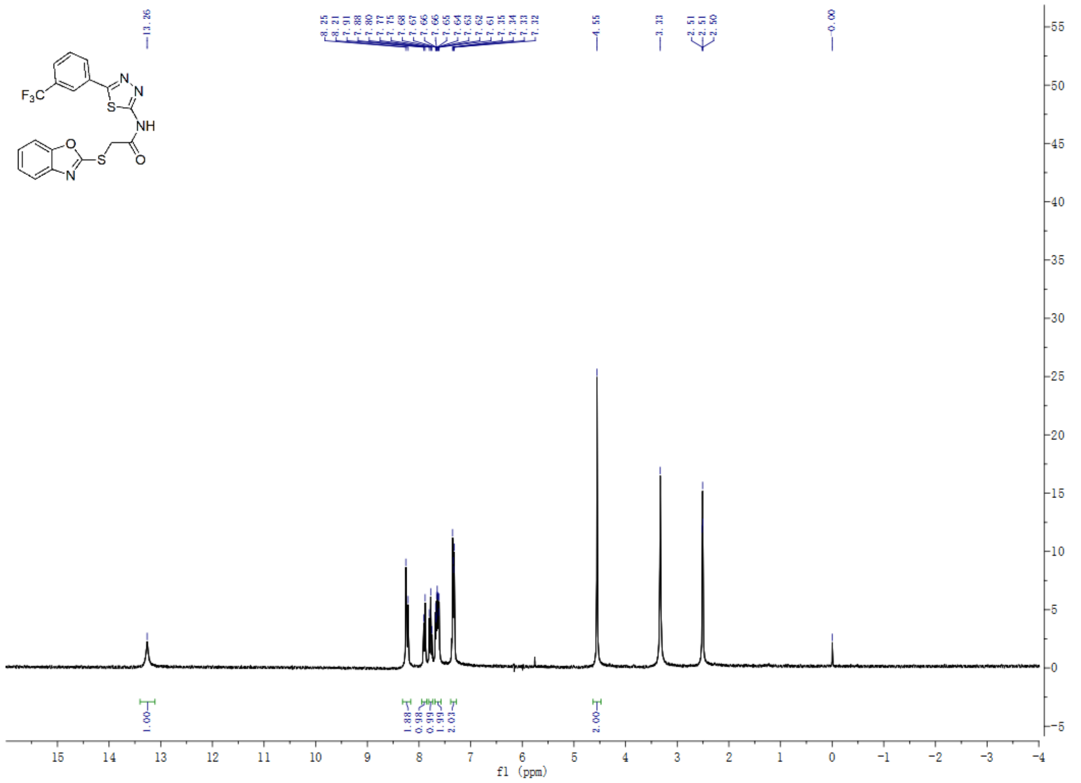

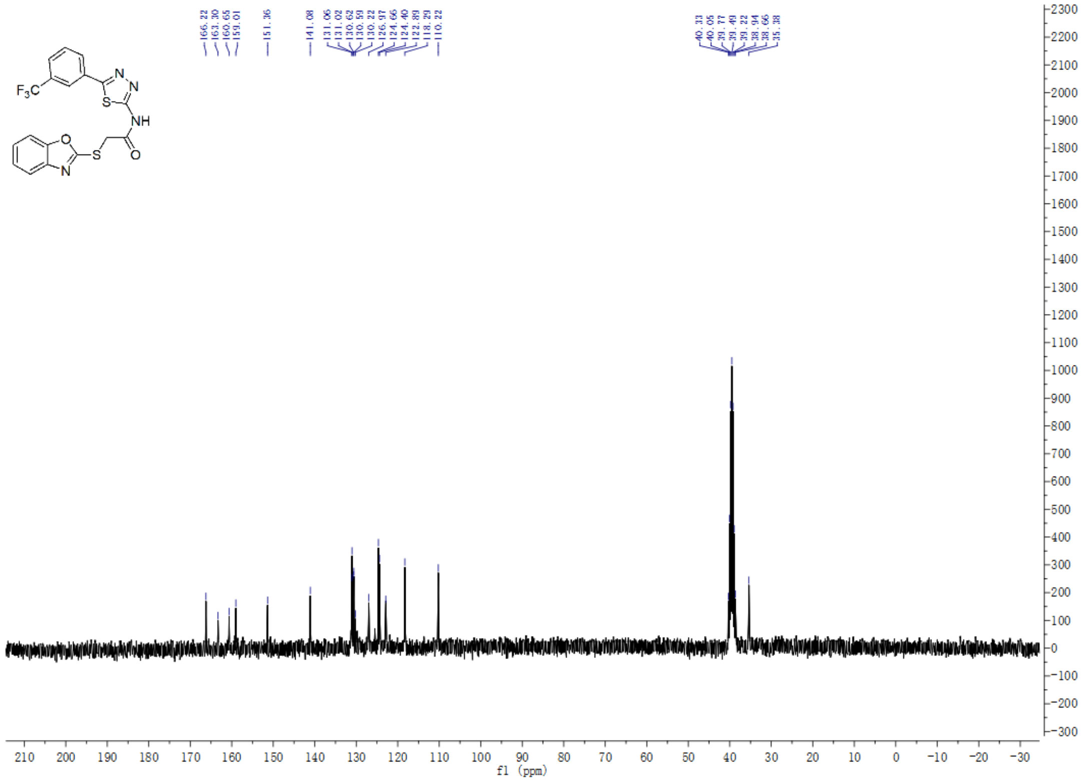

^{1}H NMR and ^{13}C NMR spectra of compound **5k**

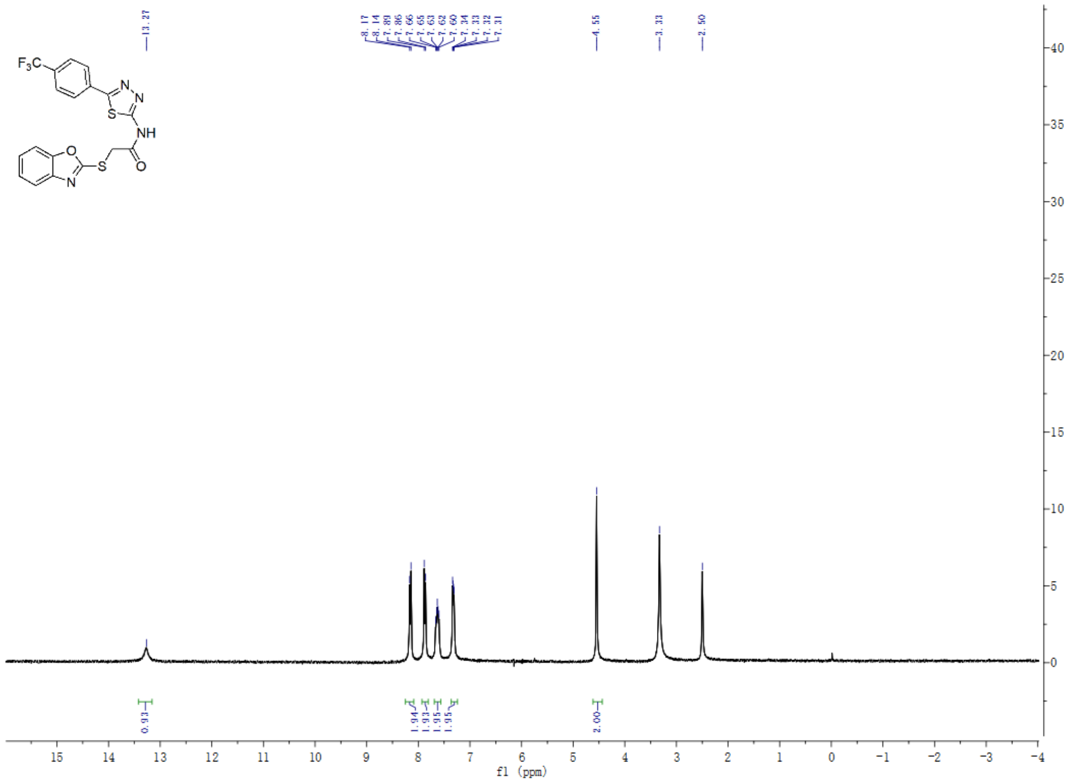

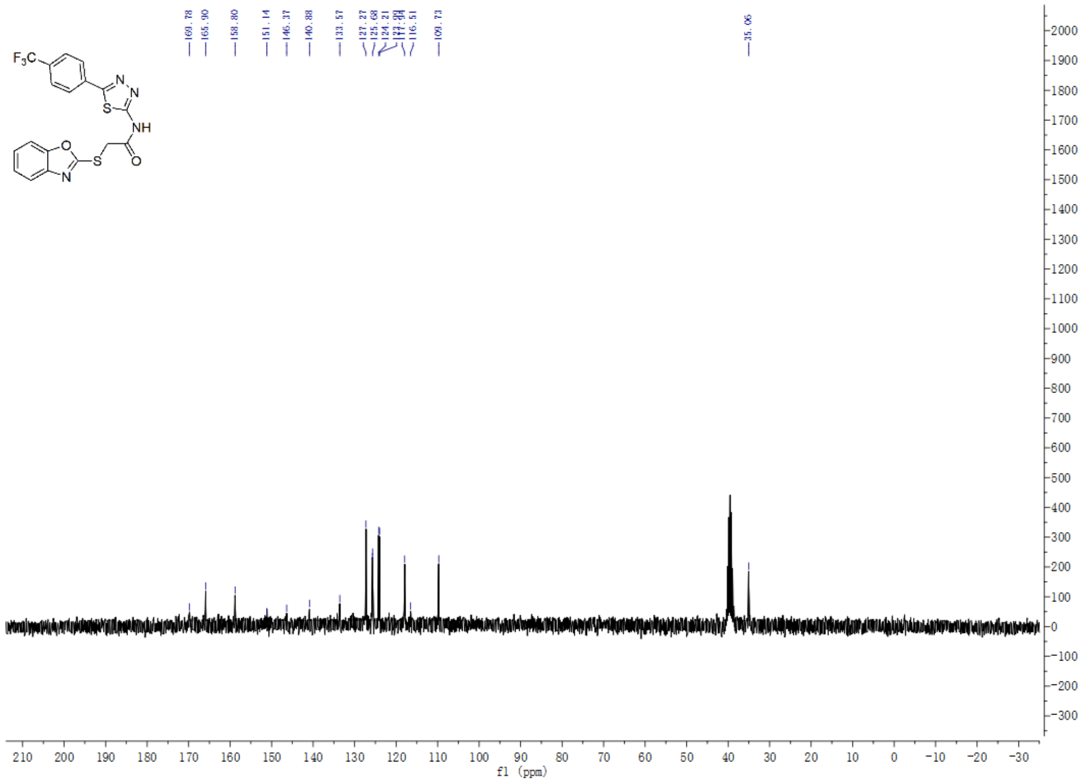

^{1}H NMR and ^{13}C NMR spectra of compound **5l**

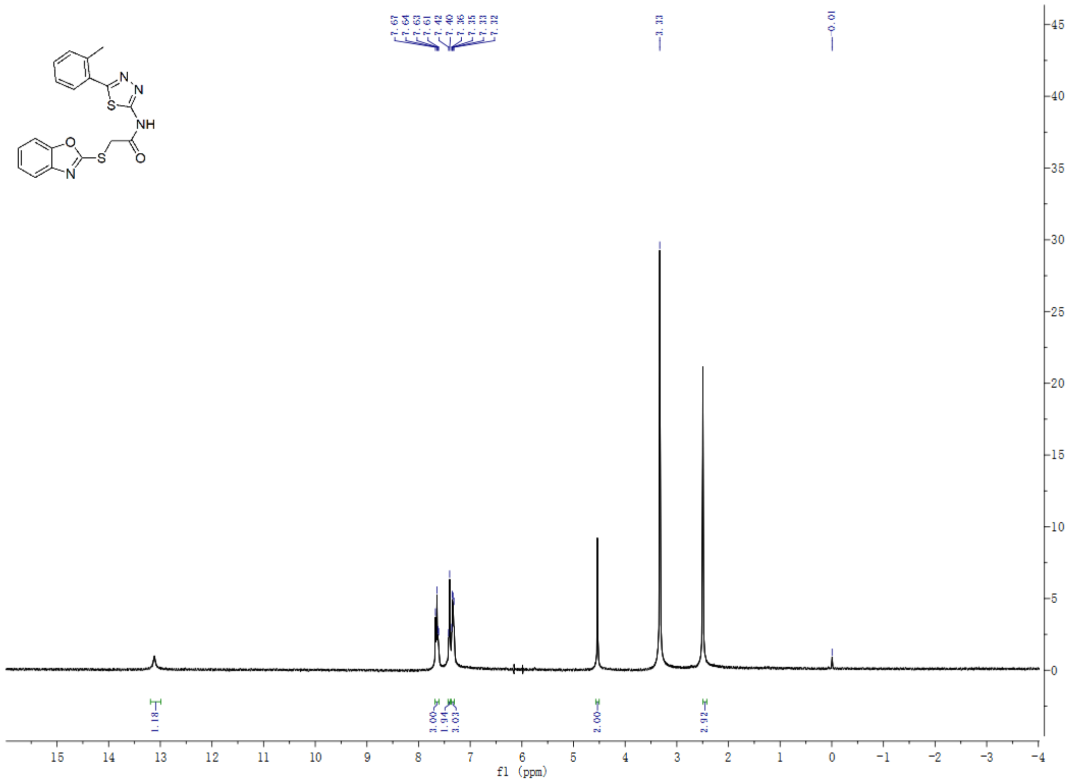

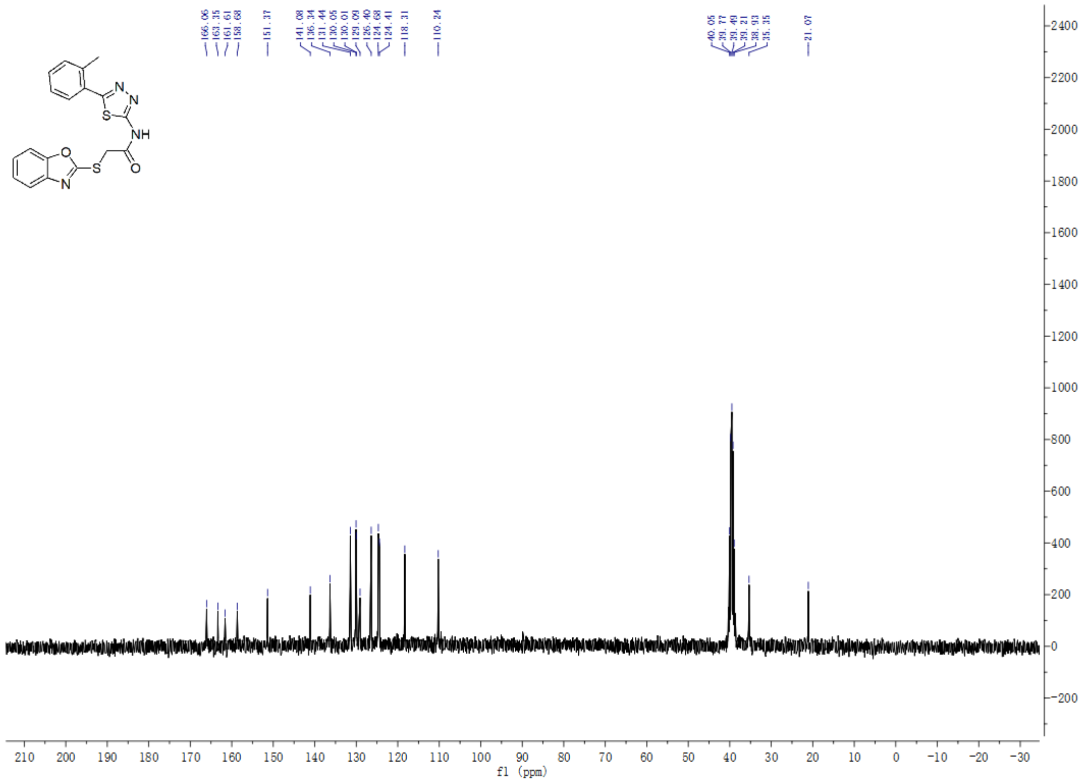

^{1}H NMR and ^{13}C NMR spectra of compound **5m**

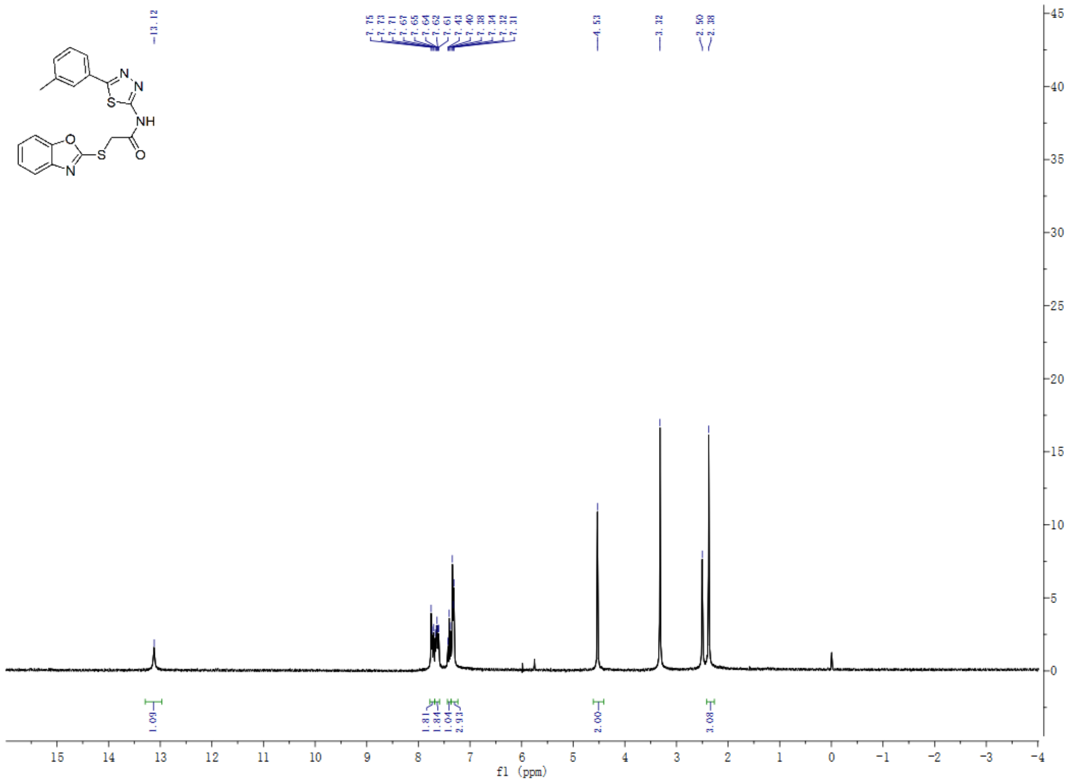

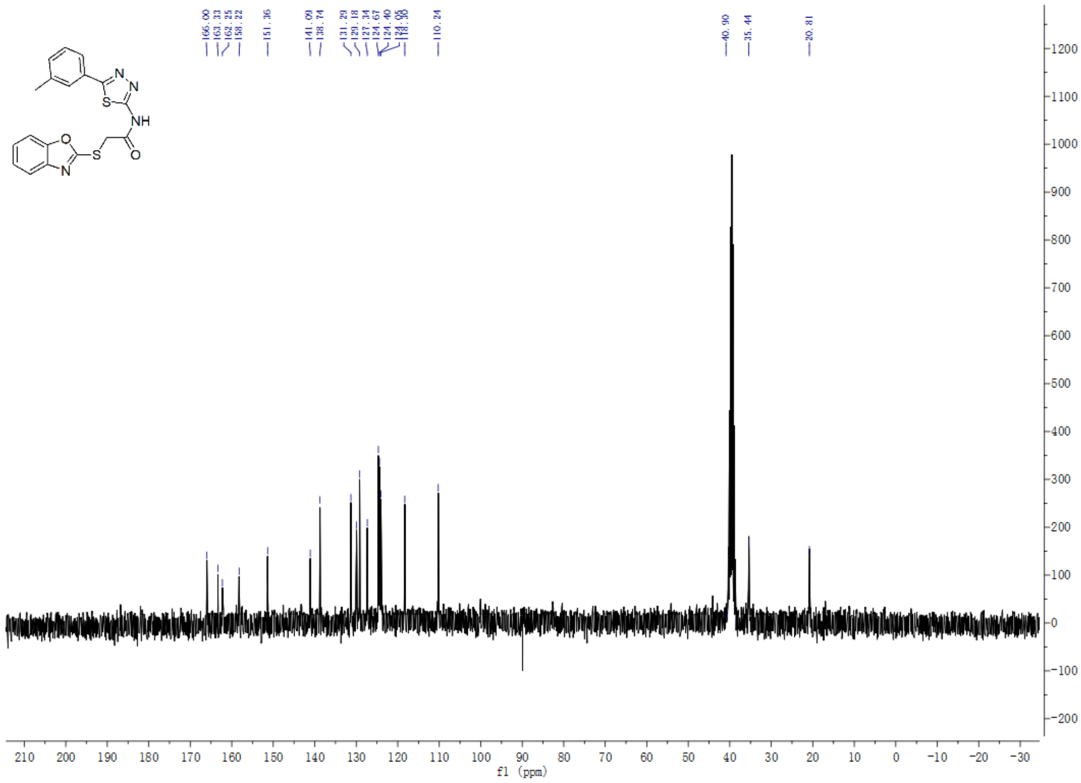

^{1}H NMR and ^{13}C NMR spectra of compound **5n**

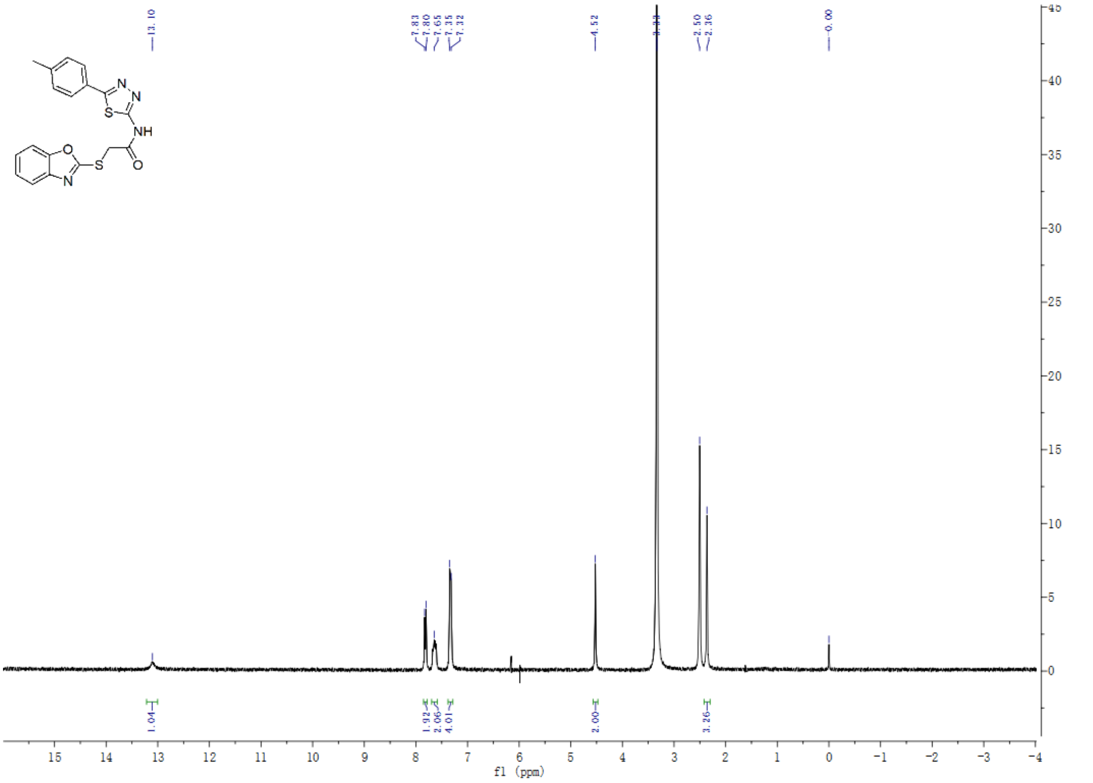

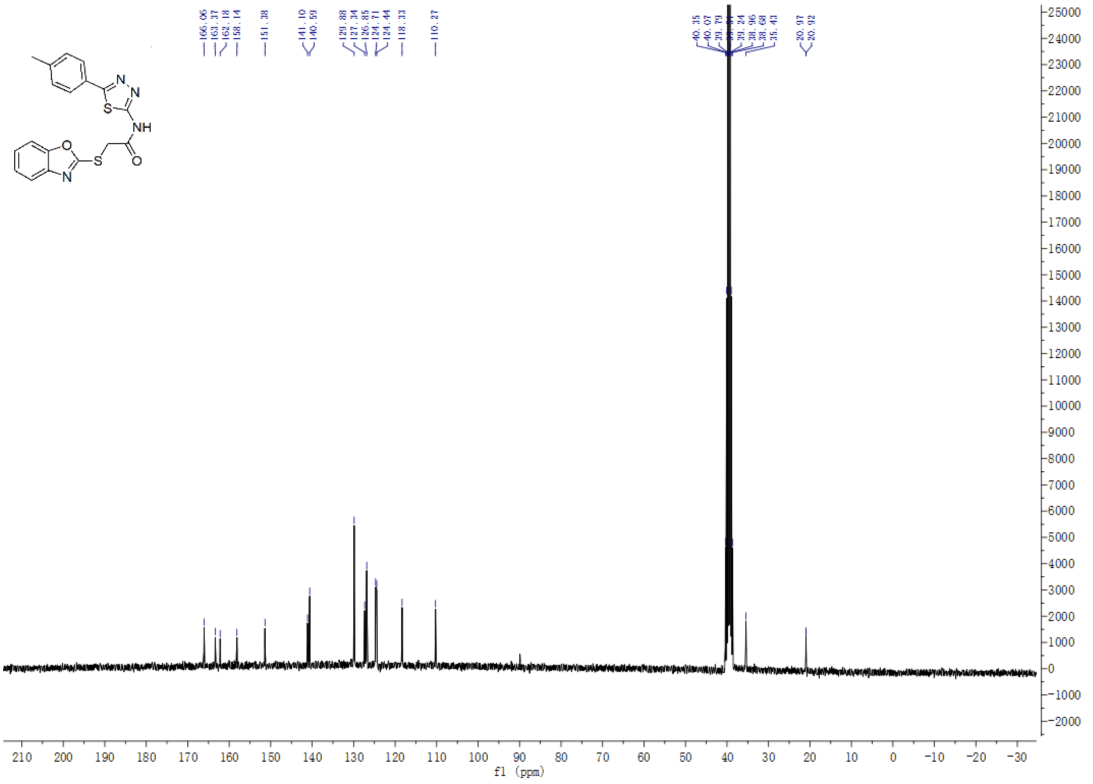

^{1}H NMR and ^{13}C NMR spectra of compound **5o**

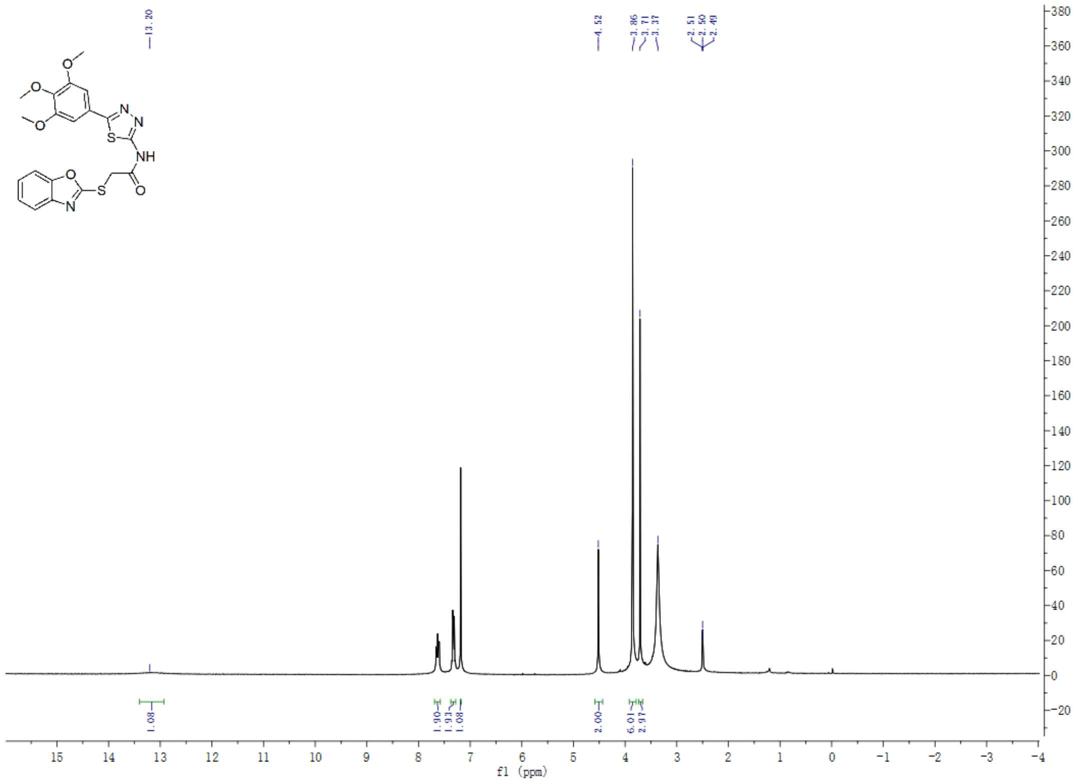

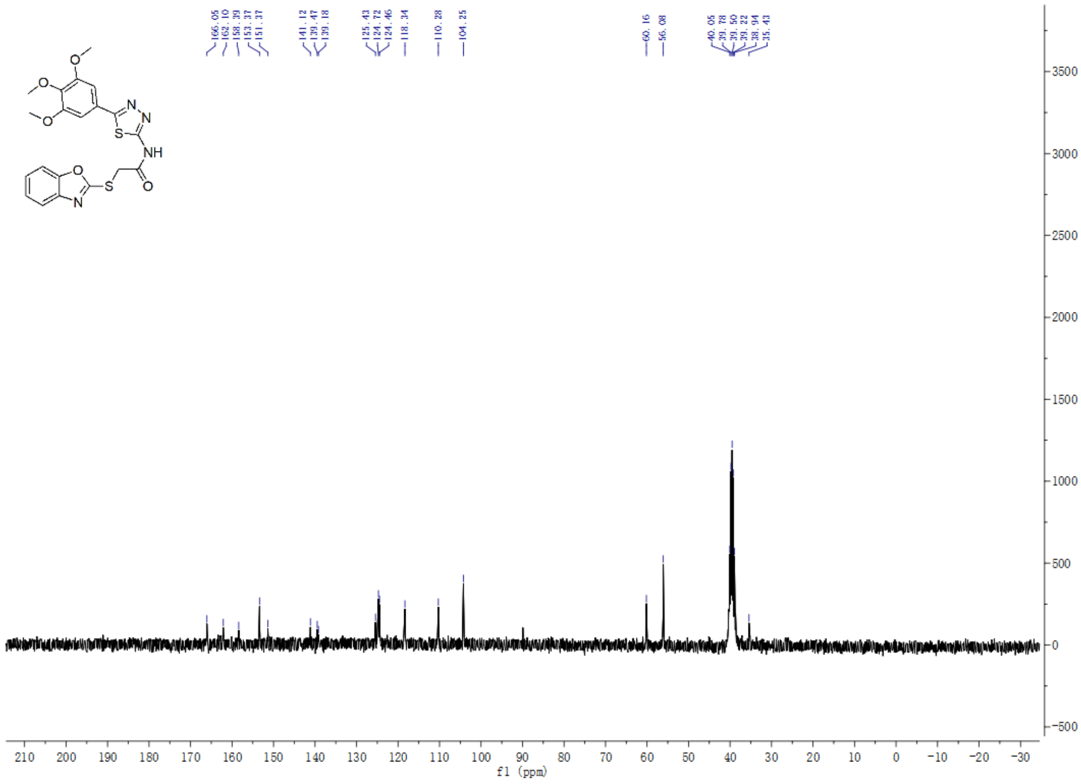

^{1}H NMR and ^{13}C NMR spectra of compound **5p**

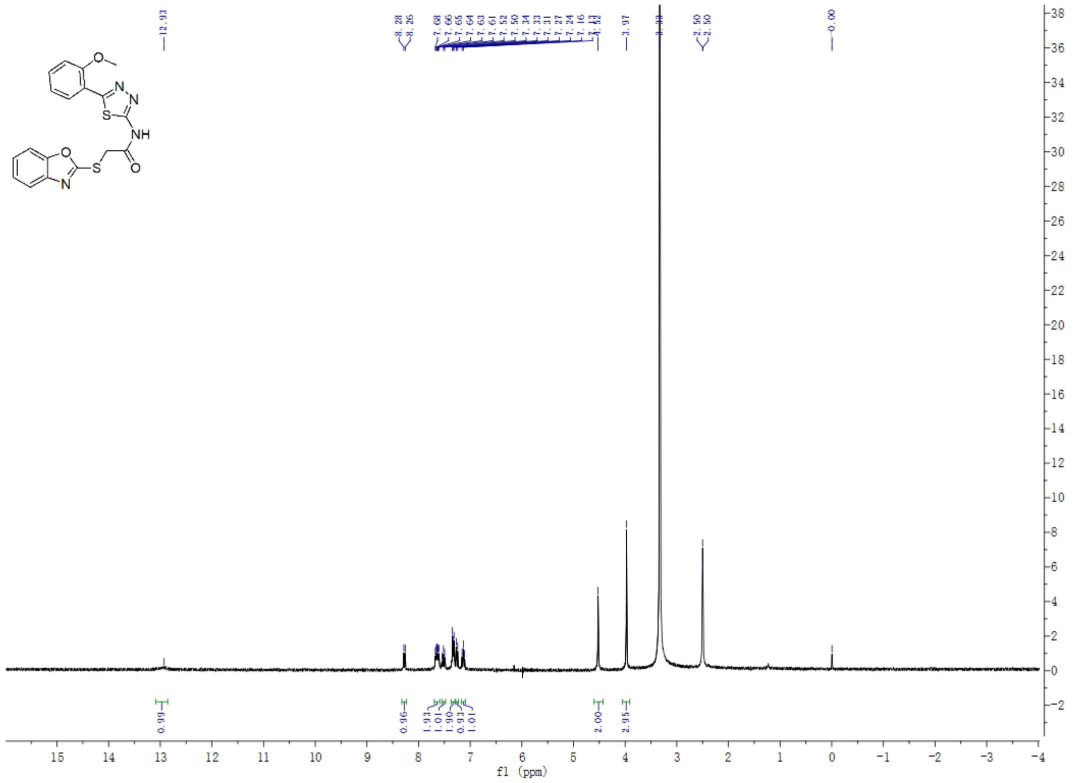

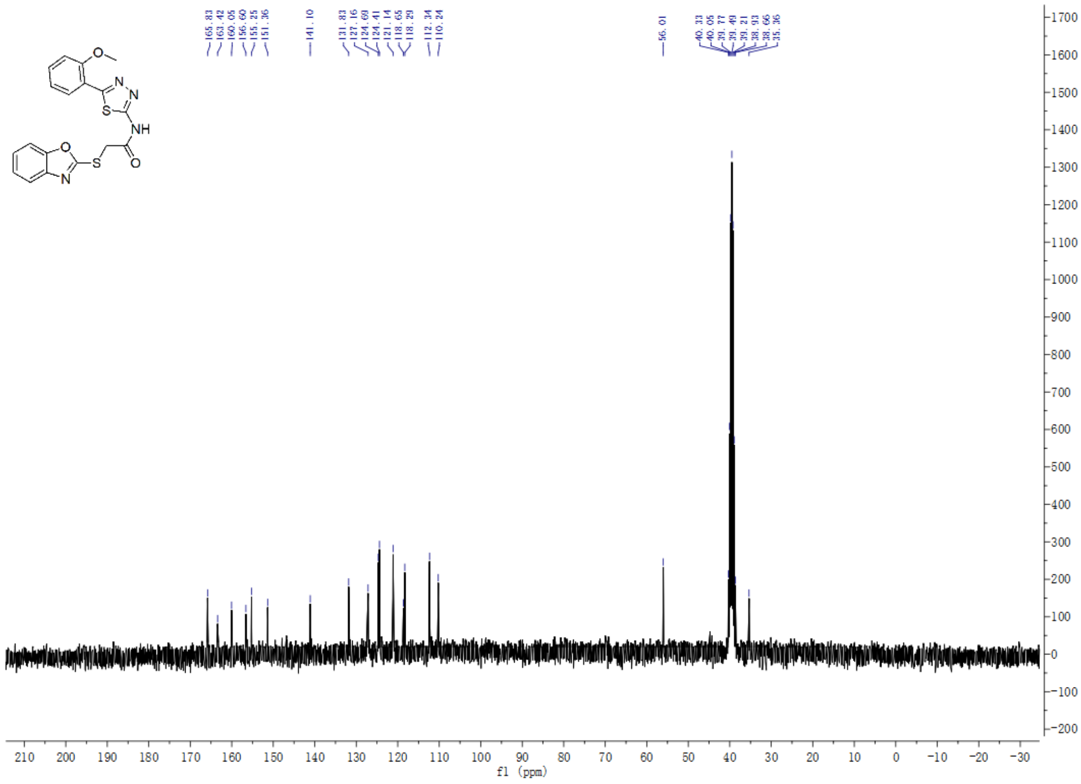

^{1}H NMR and ^{13}C NMR spectra of compound **5q**

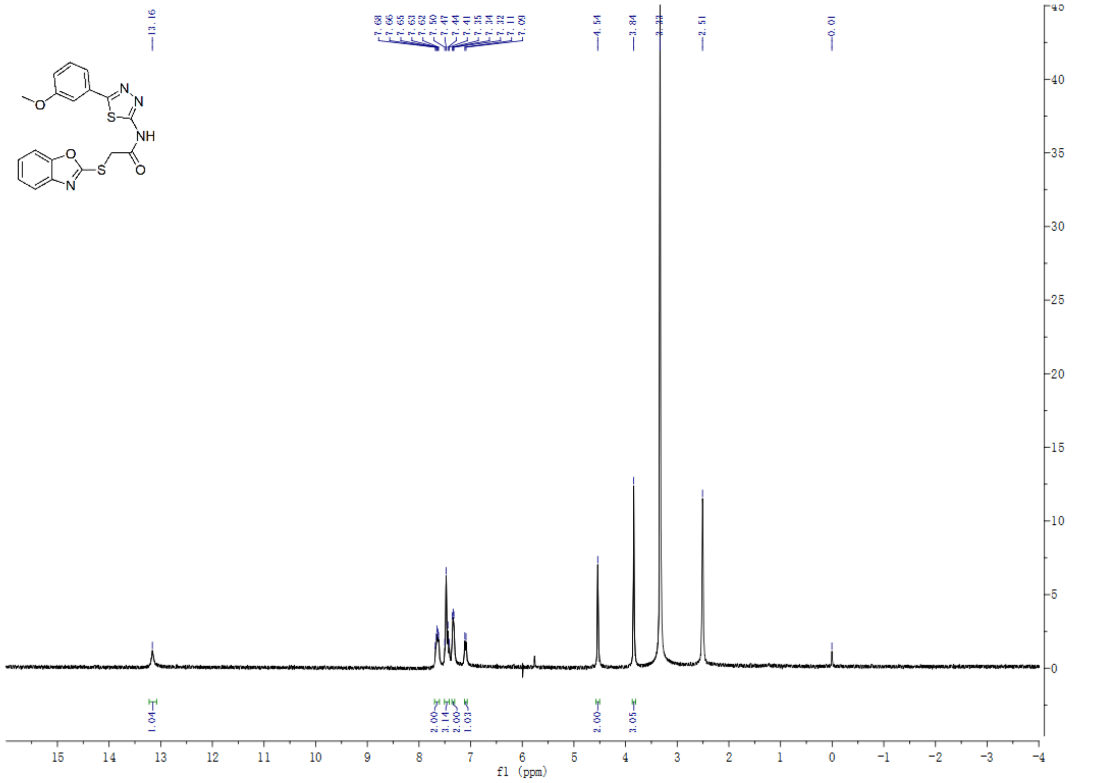

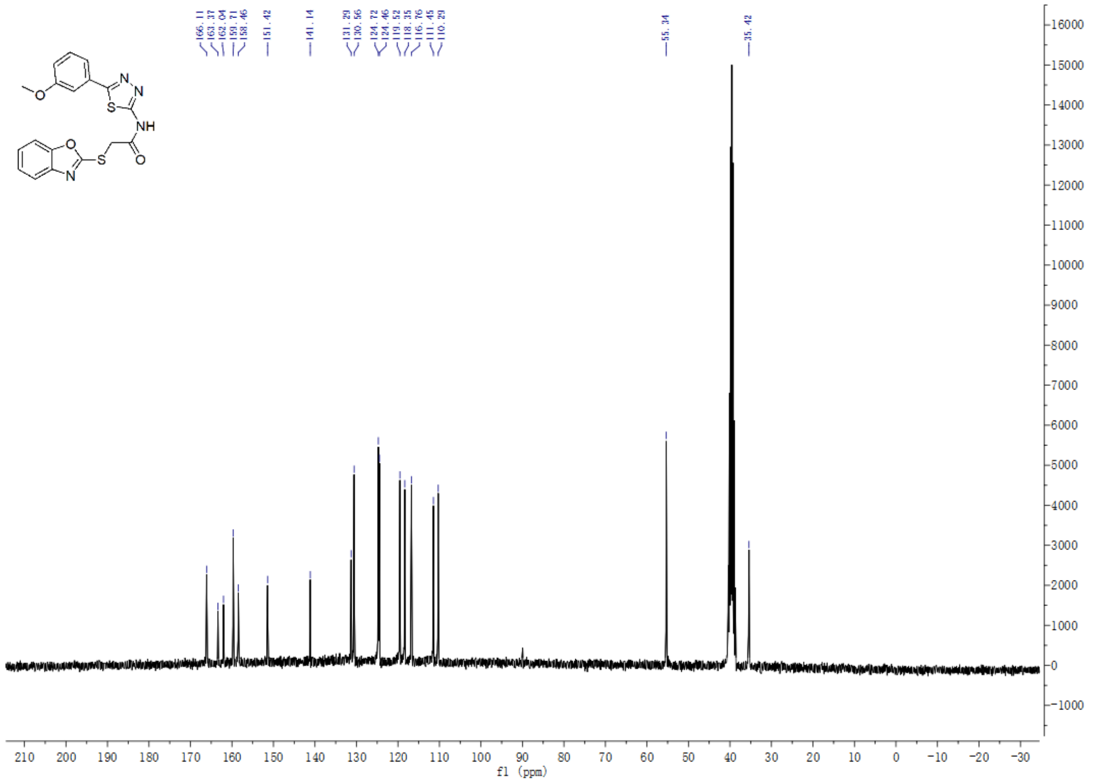

^{1}H NMR and ^{13}C NMR spectra of compound **5r**

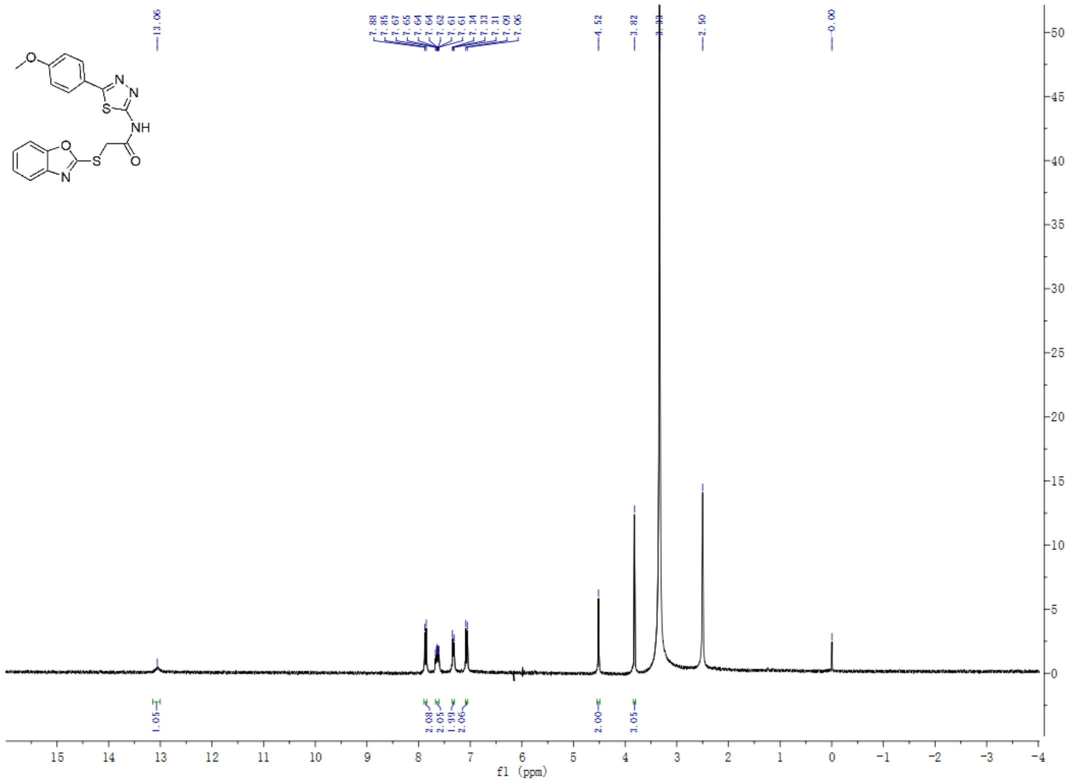

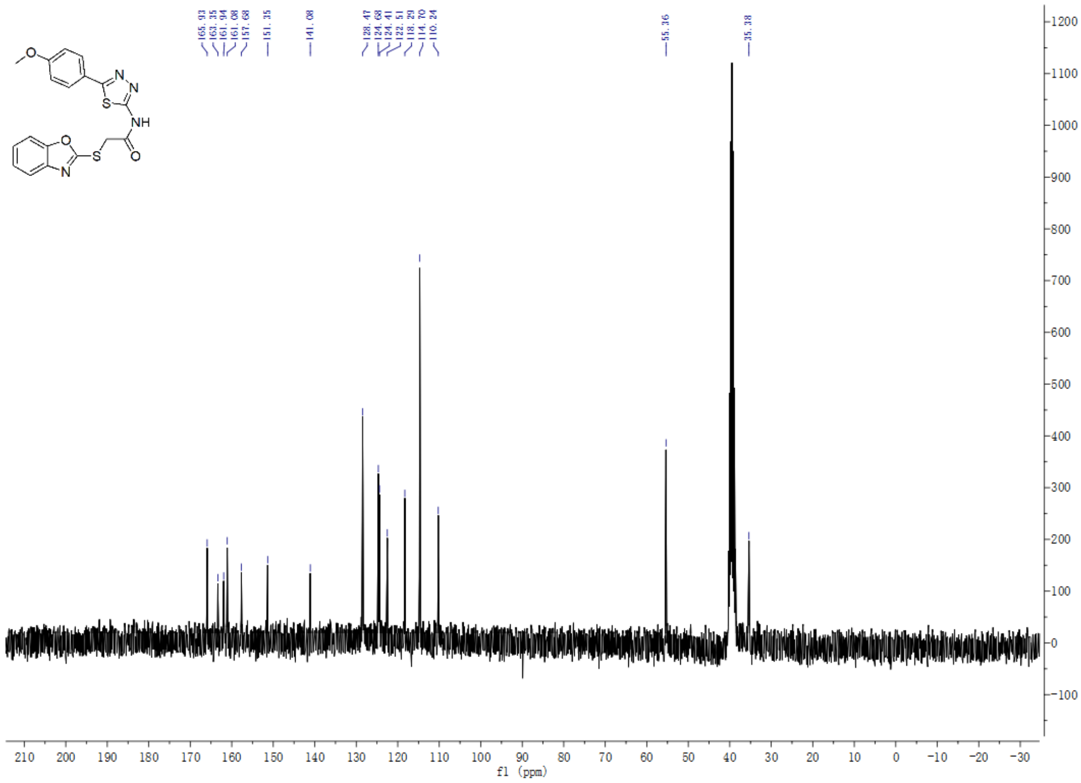

^{1}H NMR and ^{13}C NMR spectra of compound **5s**

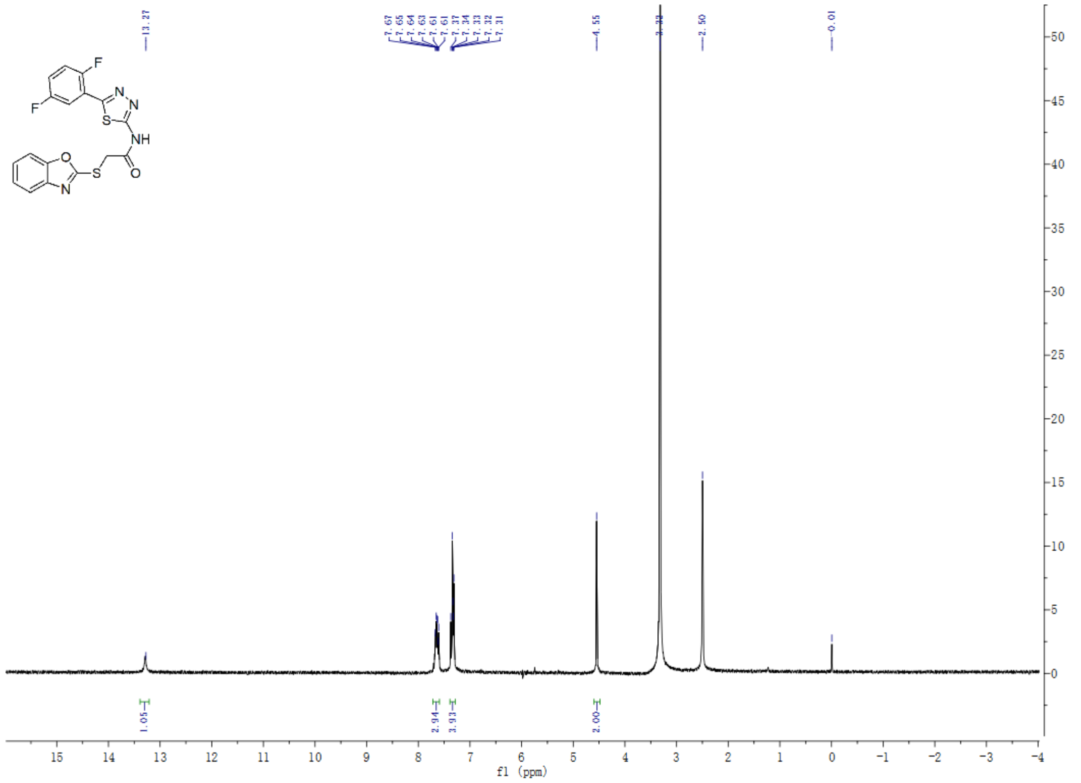

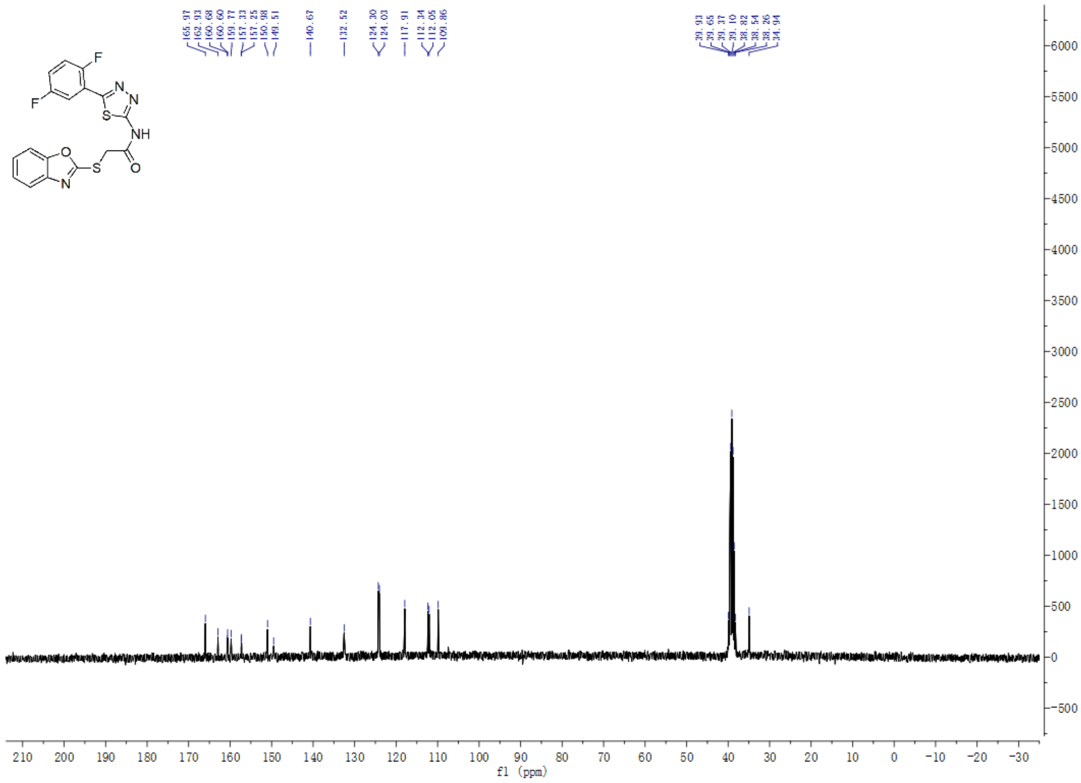

^{1}H NMR and ^{13}C NMR spectra of compound **5t**

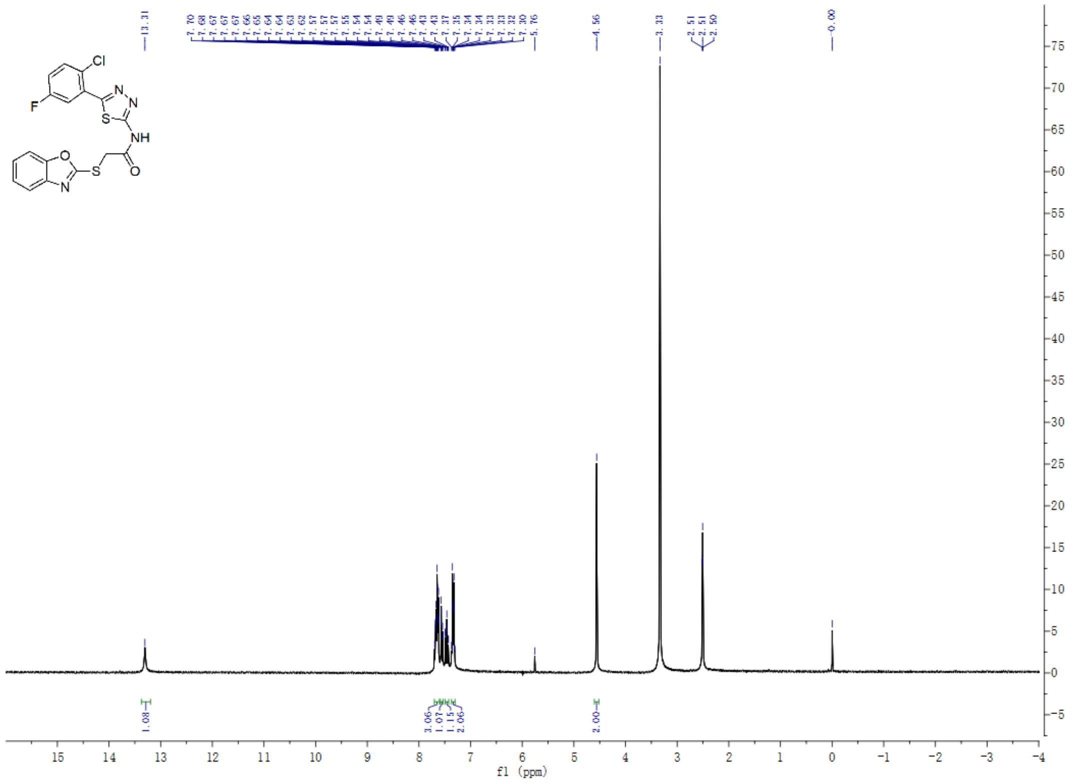

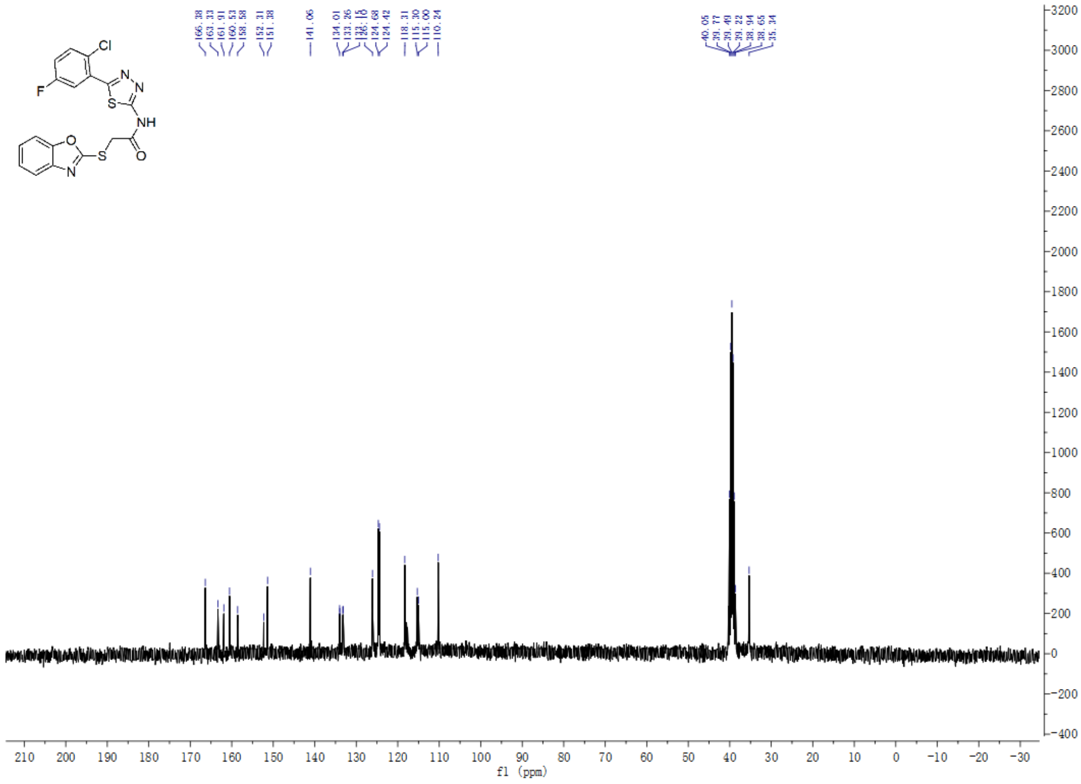

^{1}H NMR and ^{13}C NMR spectra of compound **5u**

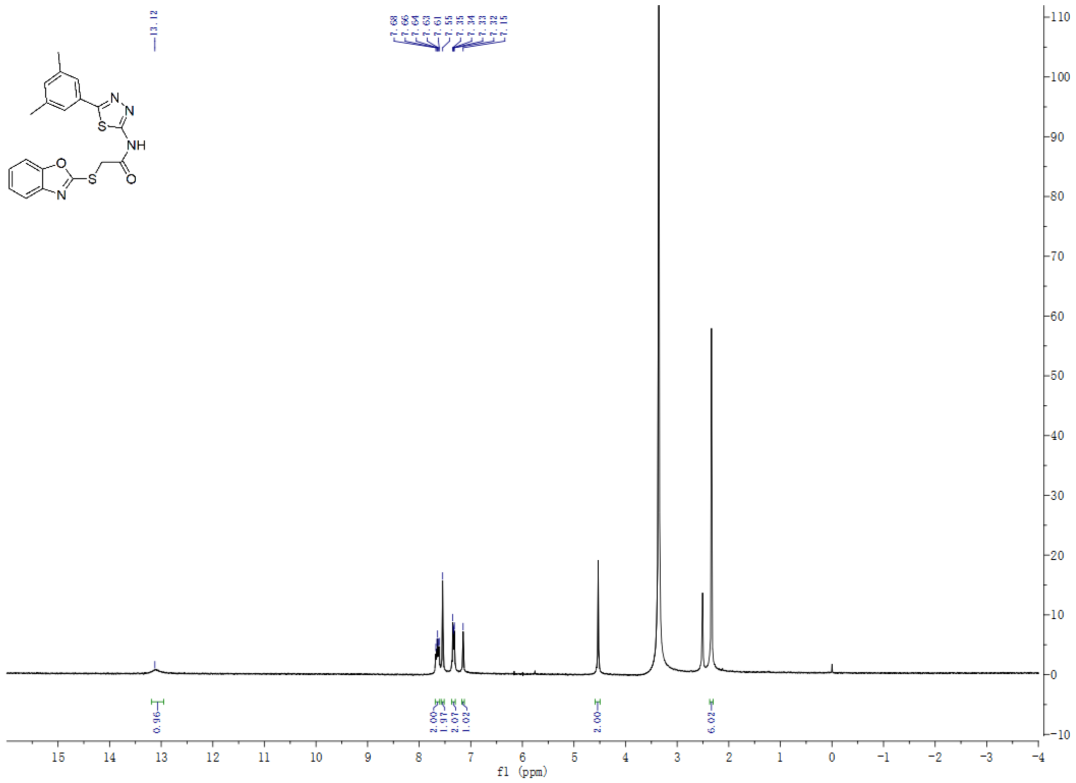

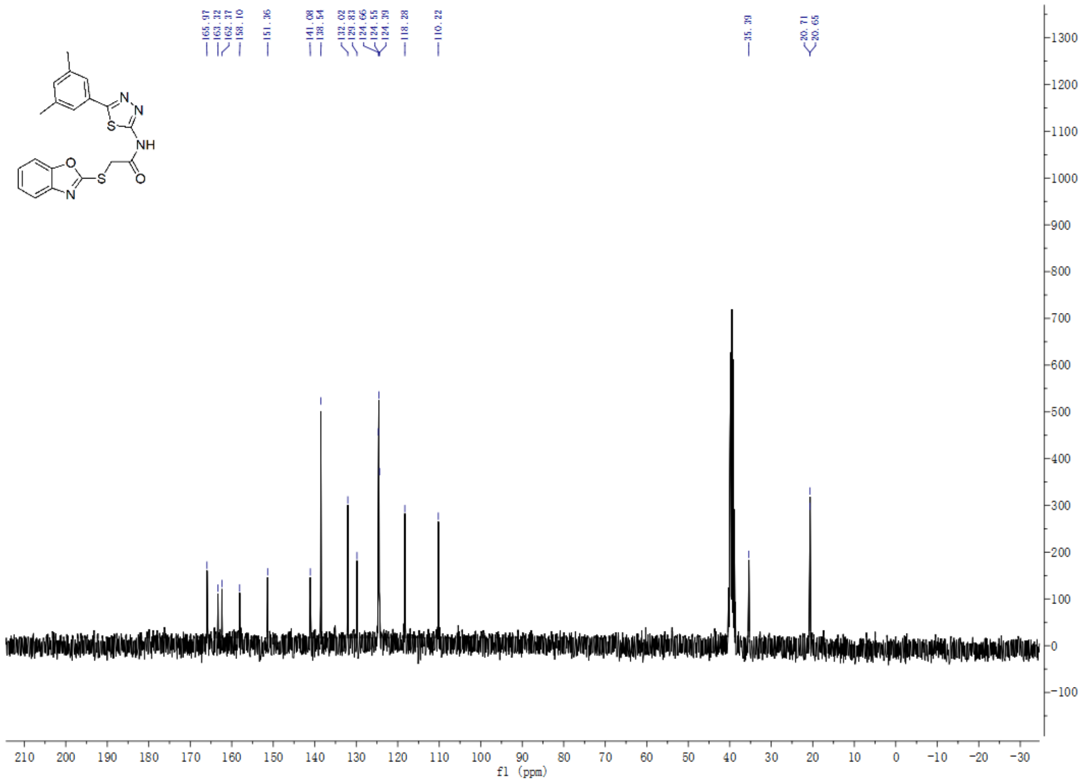

^{1}H NMR and ^{13}C NMR spectra of compound **5v**

2. HRMS spectra of target compounds

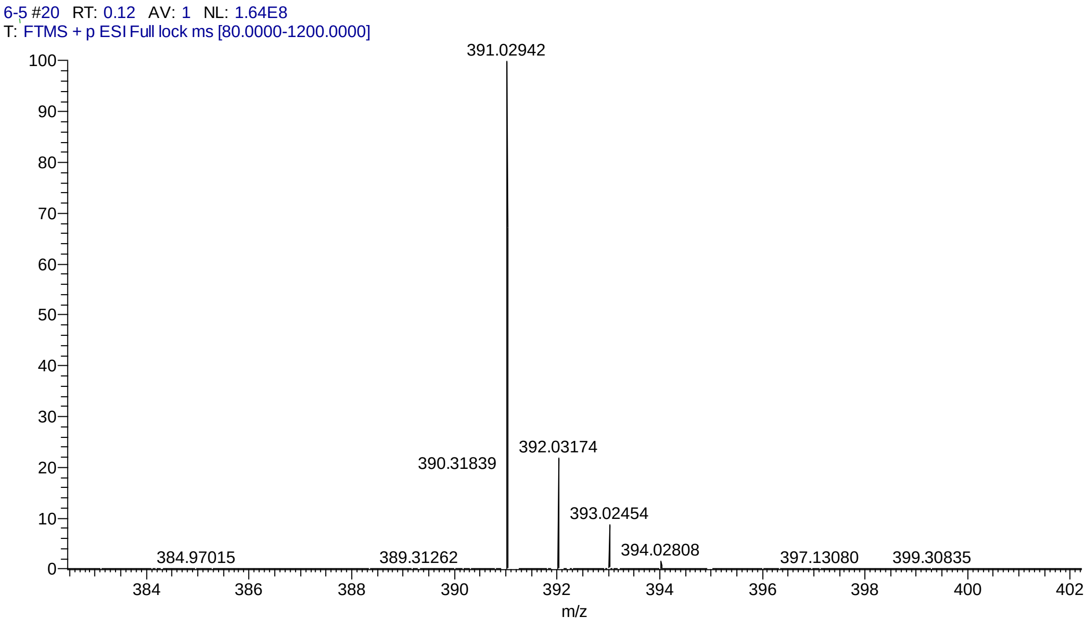

HRMS spectra of target compound **5a**

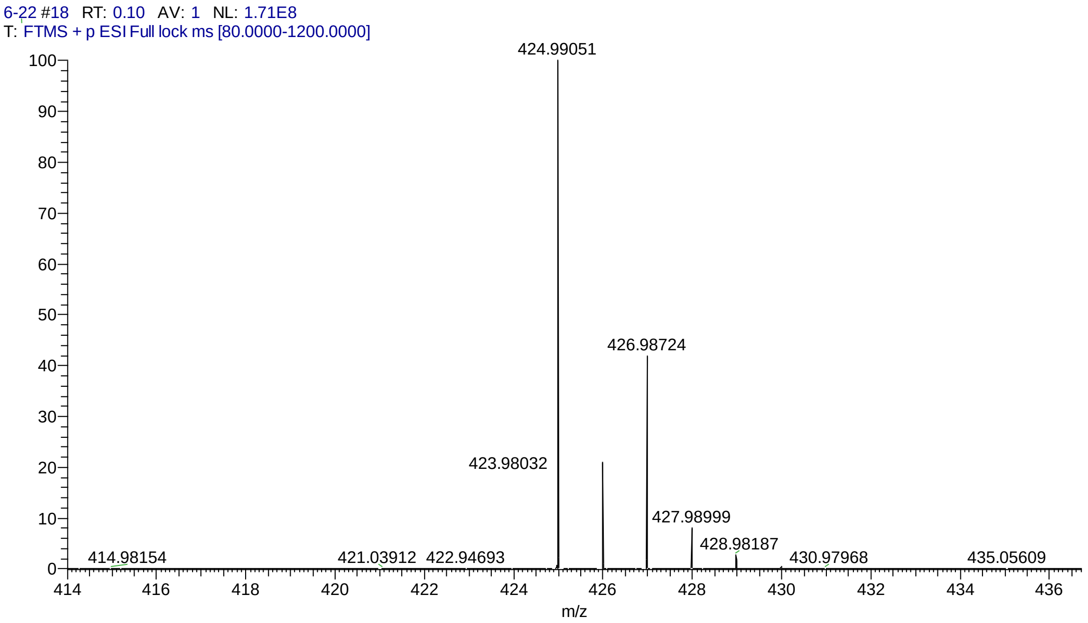

HRMS spectra of target compound **5b**

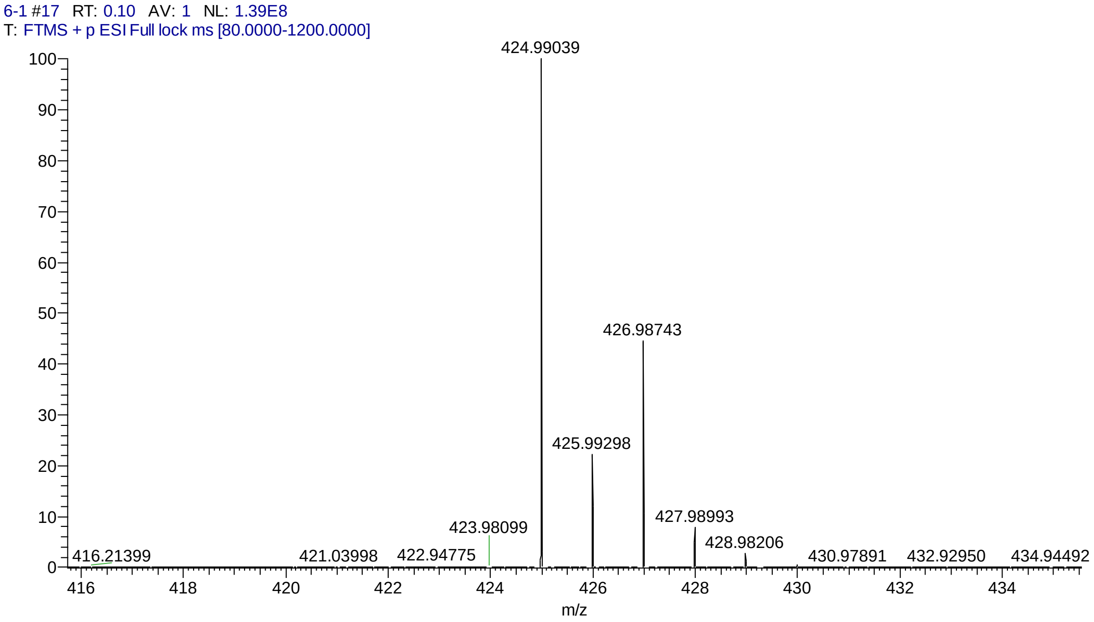

HRMS spectra of target compound **5c**

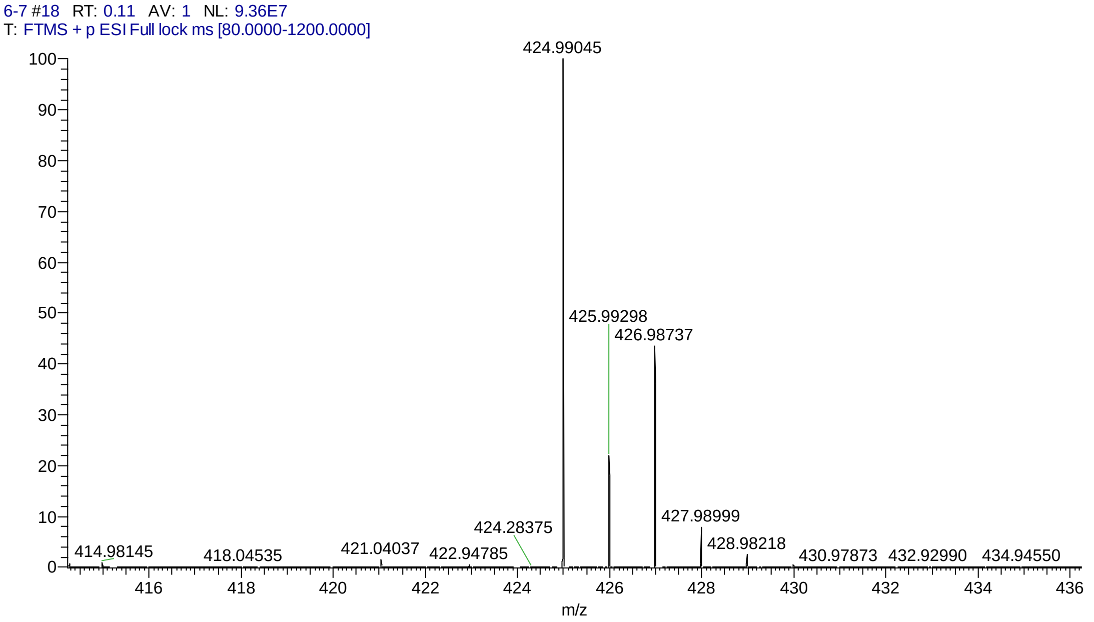

HRMS spectra of target compound **5d**

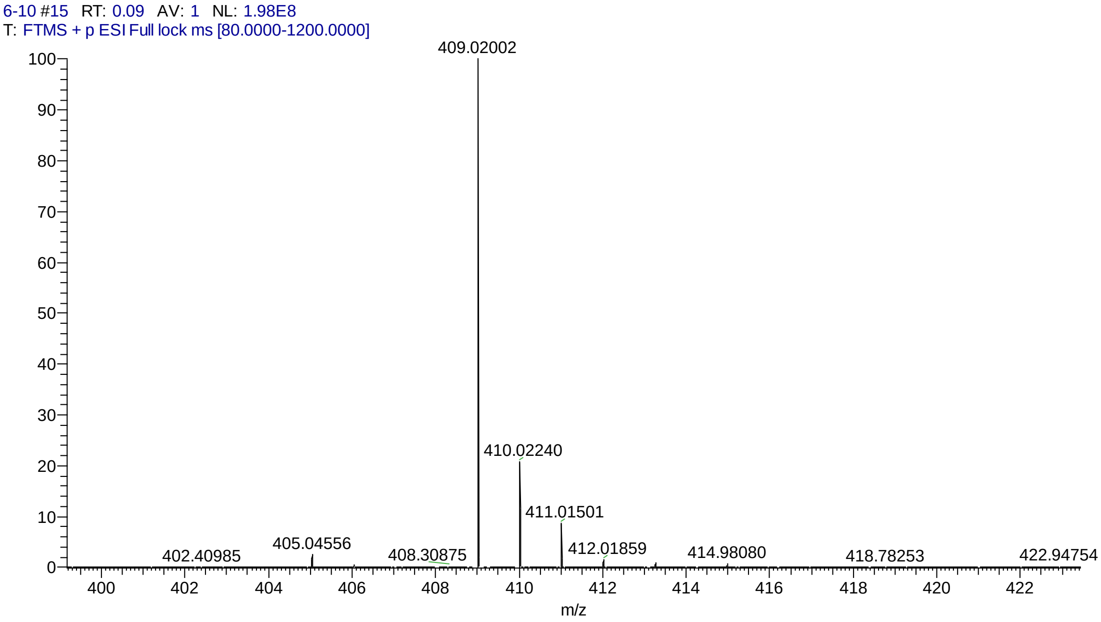

HRMS spectra of target compound **5e**

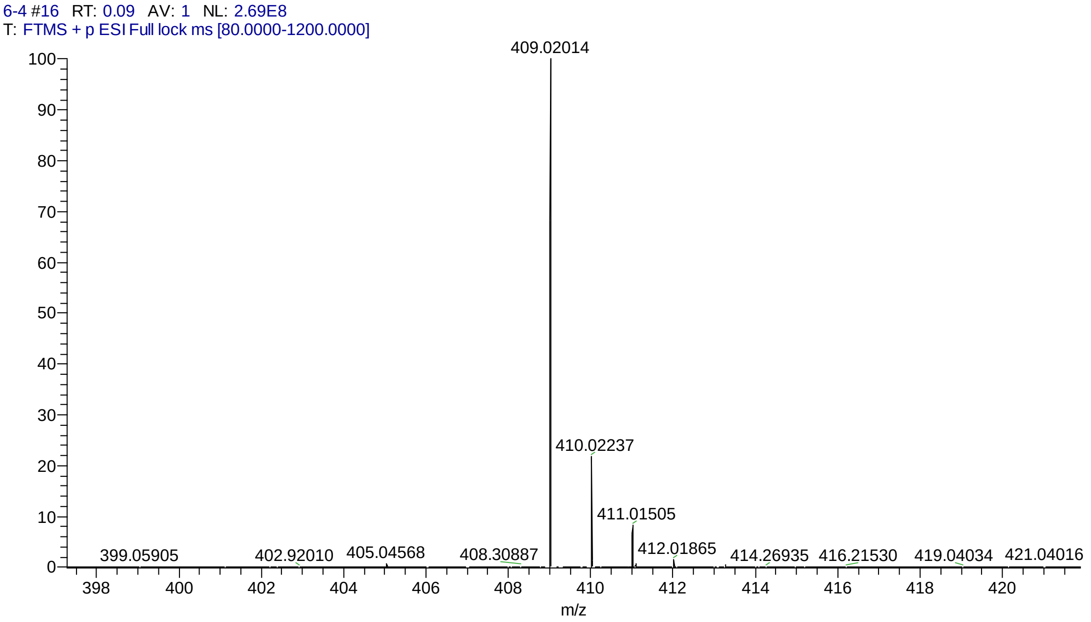

HRMS spectra of target compound **5f**

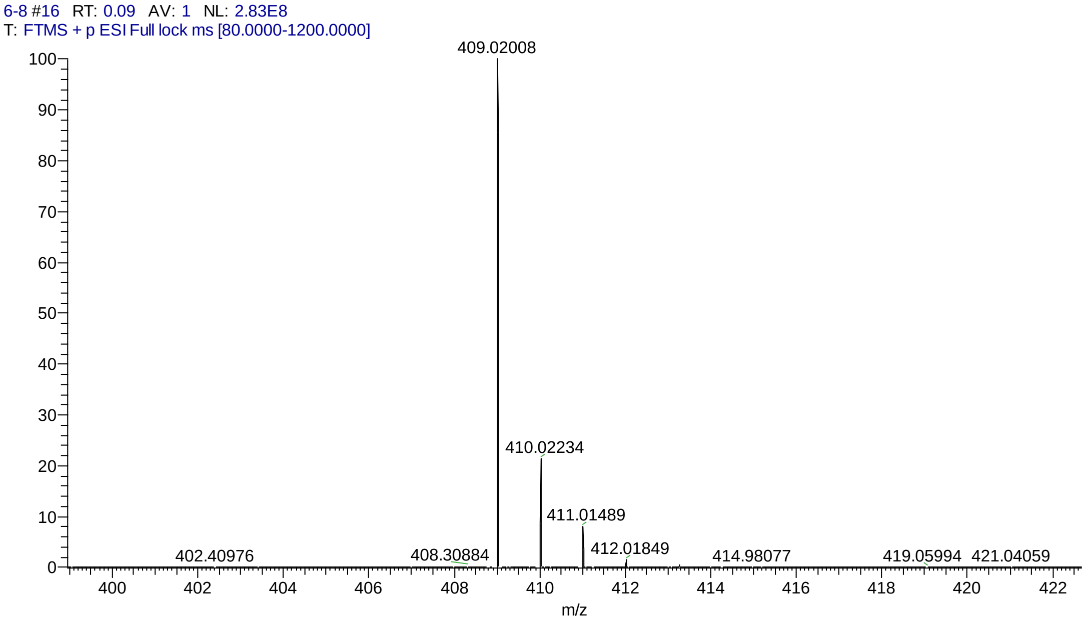

HRMS spectra of target compound **5g**

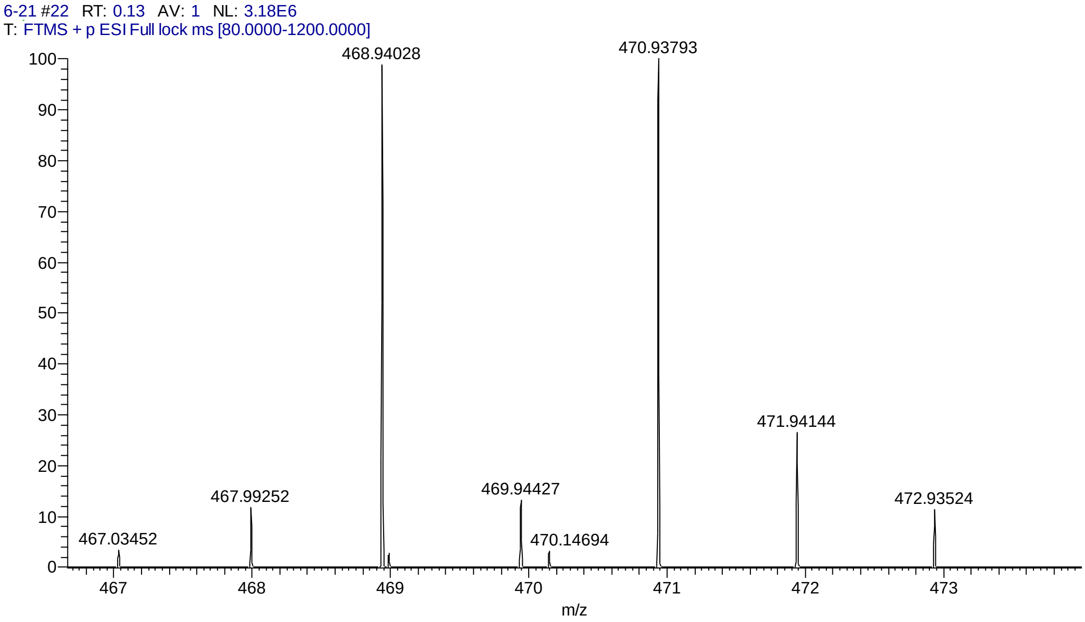

HRMS spectra of target compound **5h**

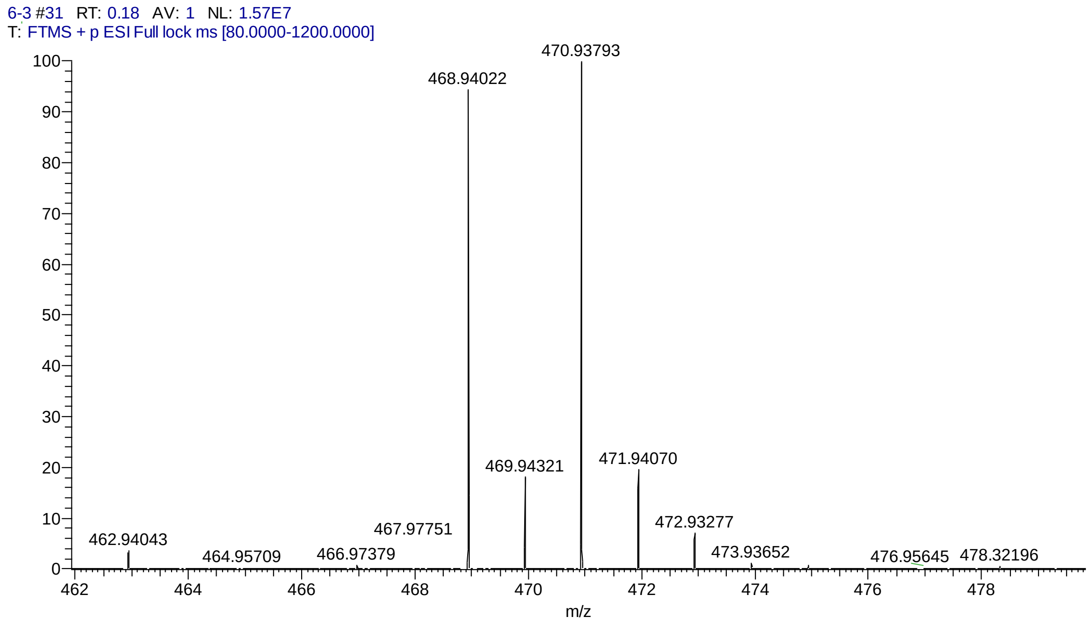

HRMS spectra of target compound **5i**

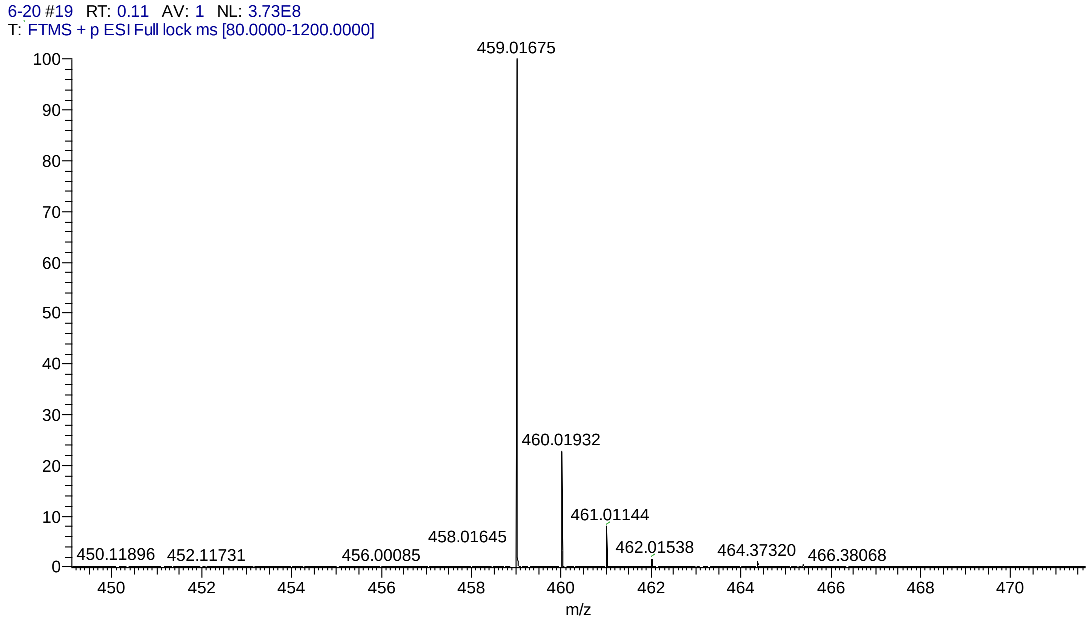

HRMS spectra of target compound **5j**

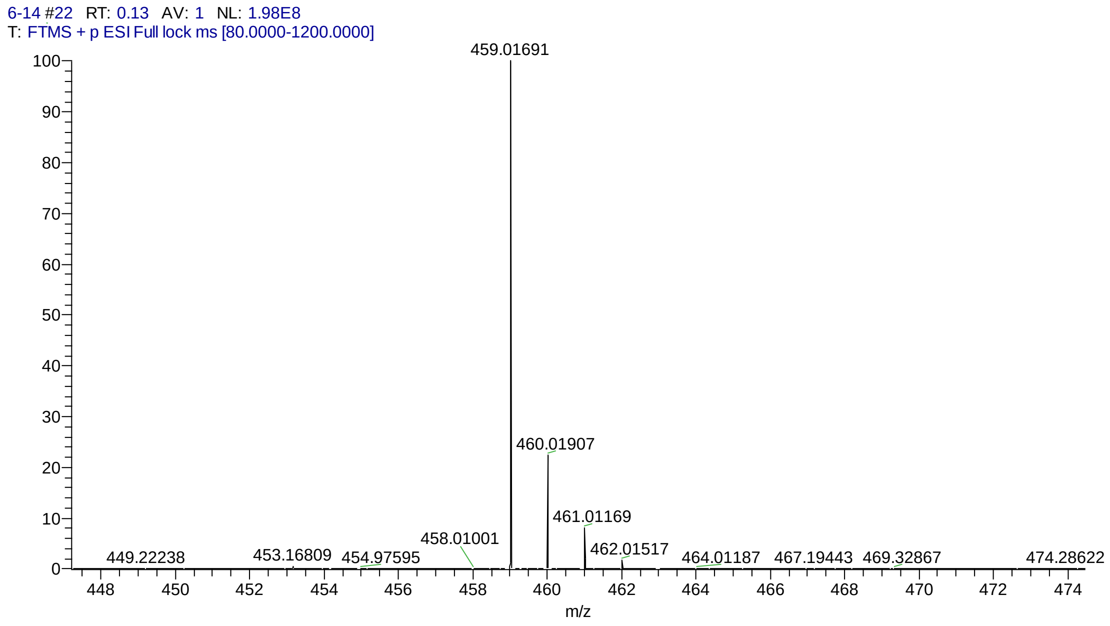

HRMS spectra of target compound **5k**

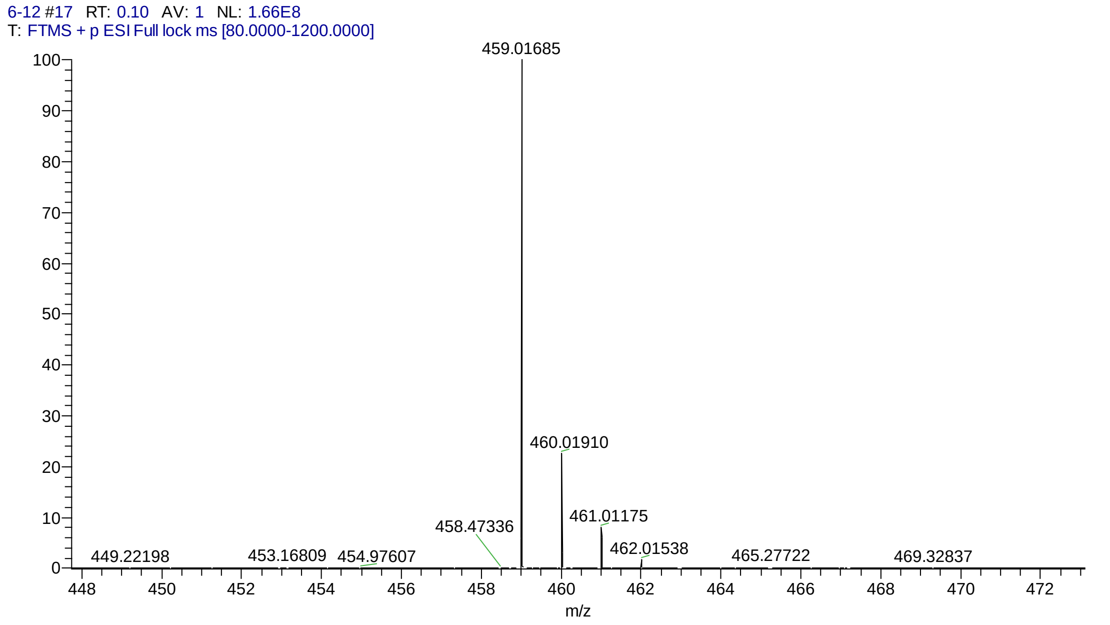

HRMS spectra of target compound **5l**

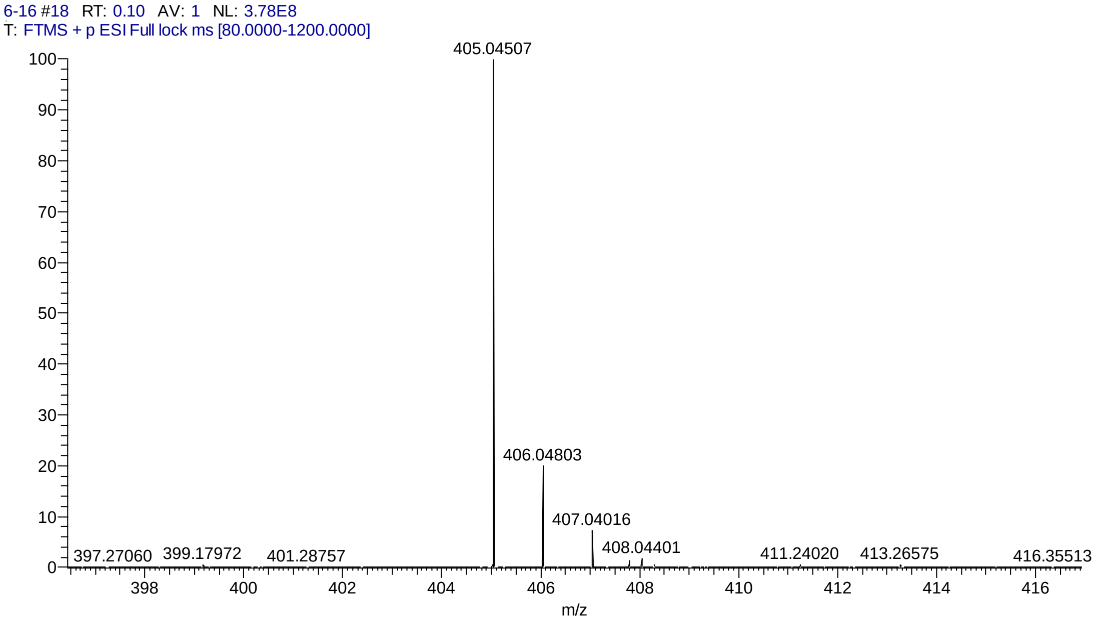

HRMS spectra of target compound **5m**

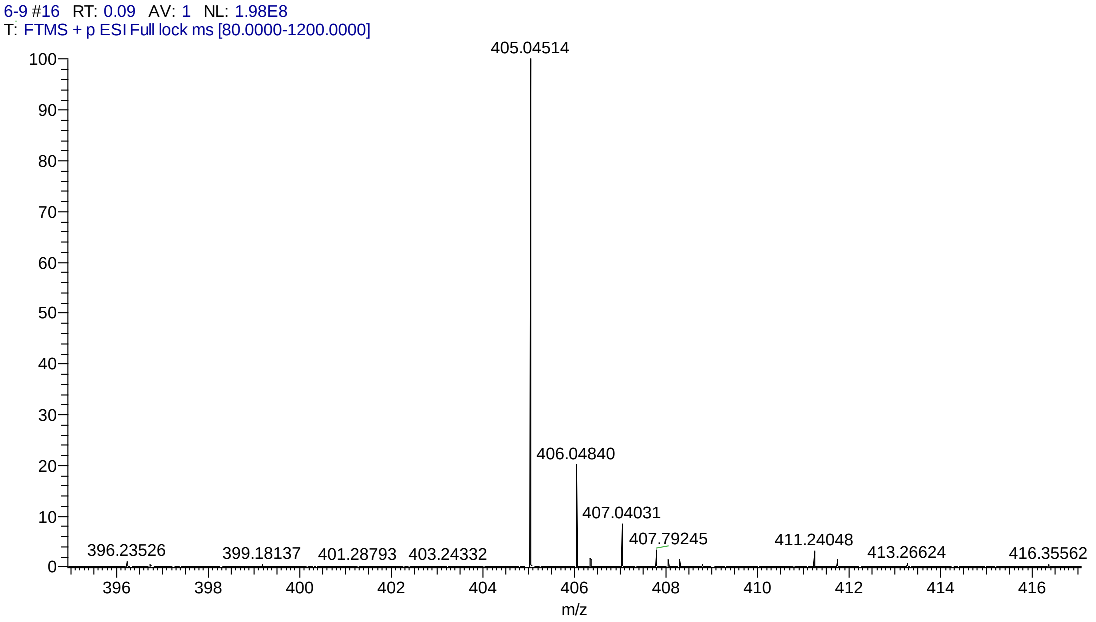

HRMS spectra of target compound **5n**

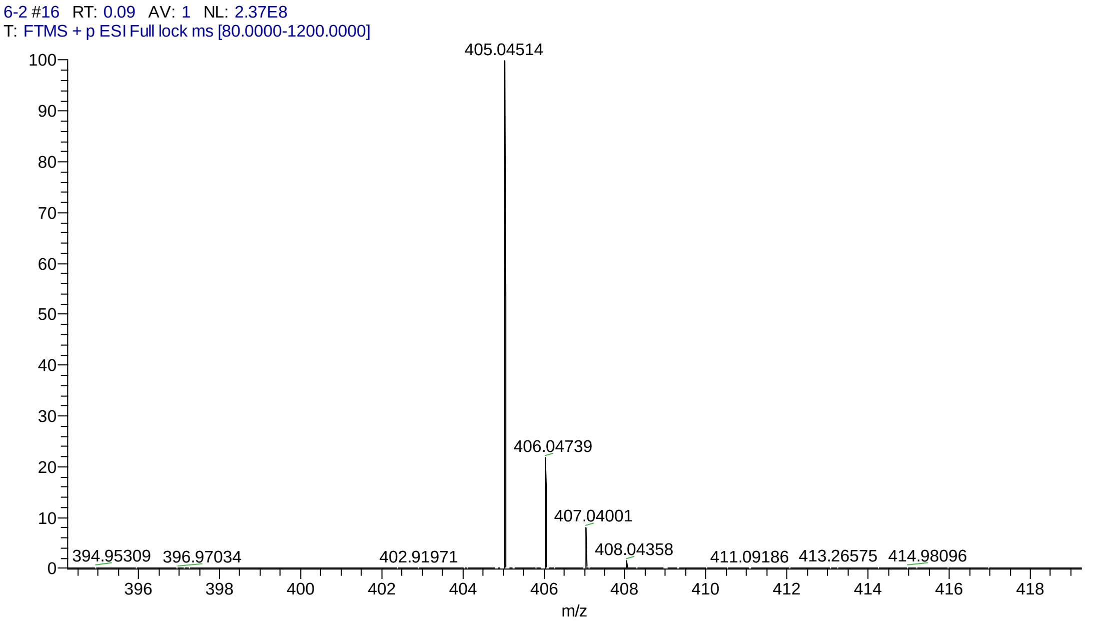

HRMS spectra of target compound **5o**

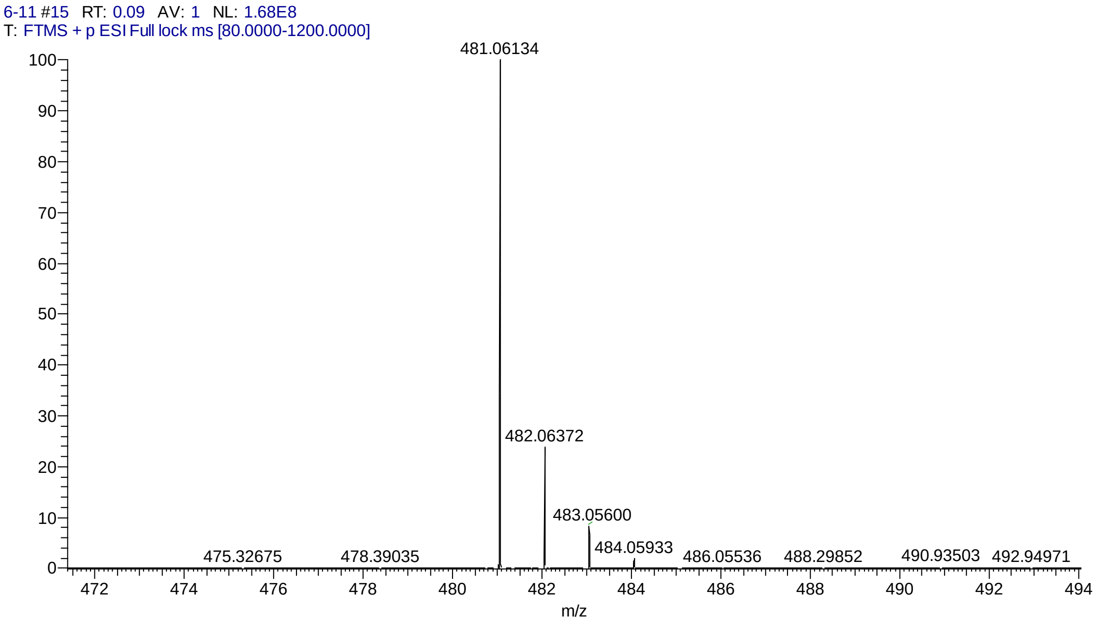

HRMS spectra of target compound **5p**

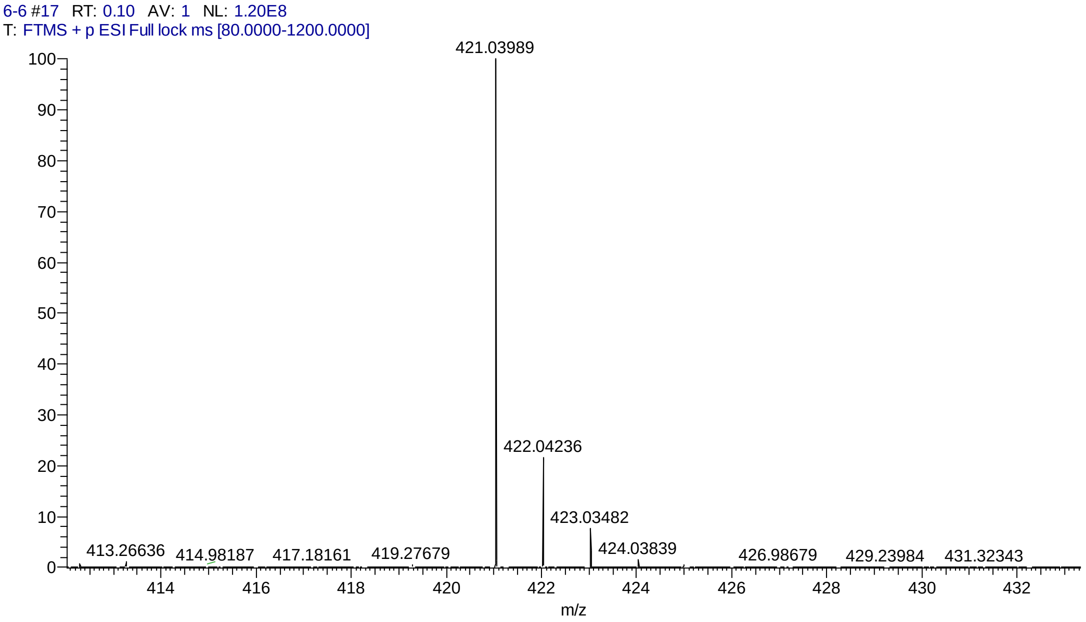

HRMS spectra of target compound **5q**

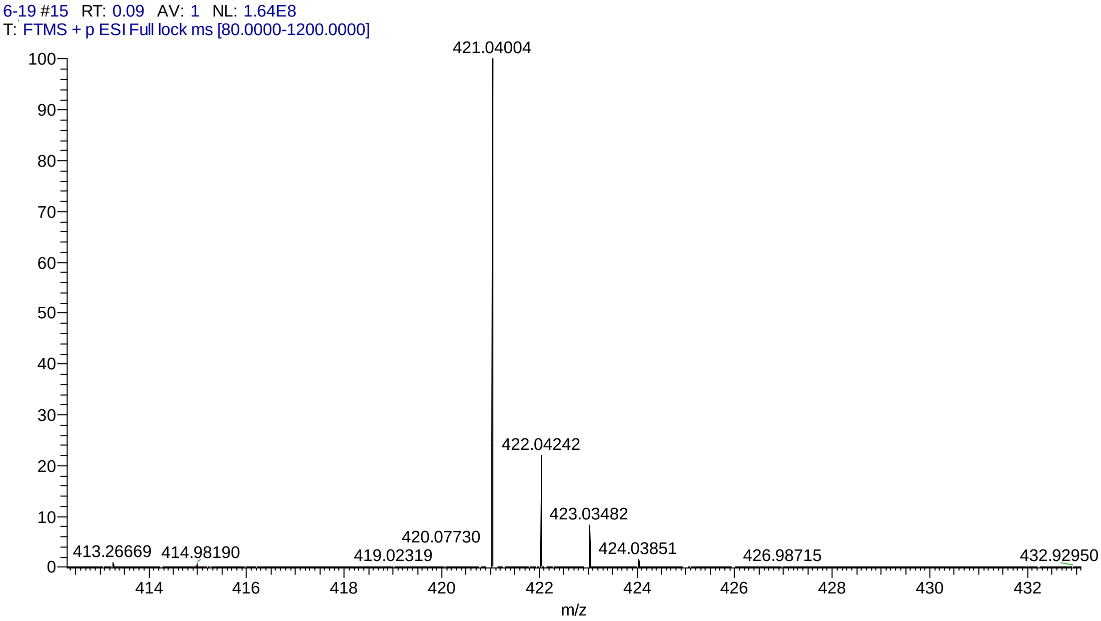

HRMS spectra of target compound **5r**

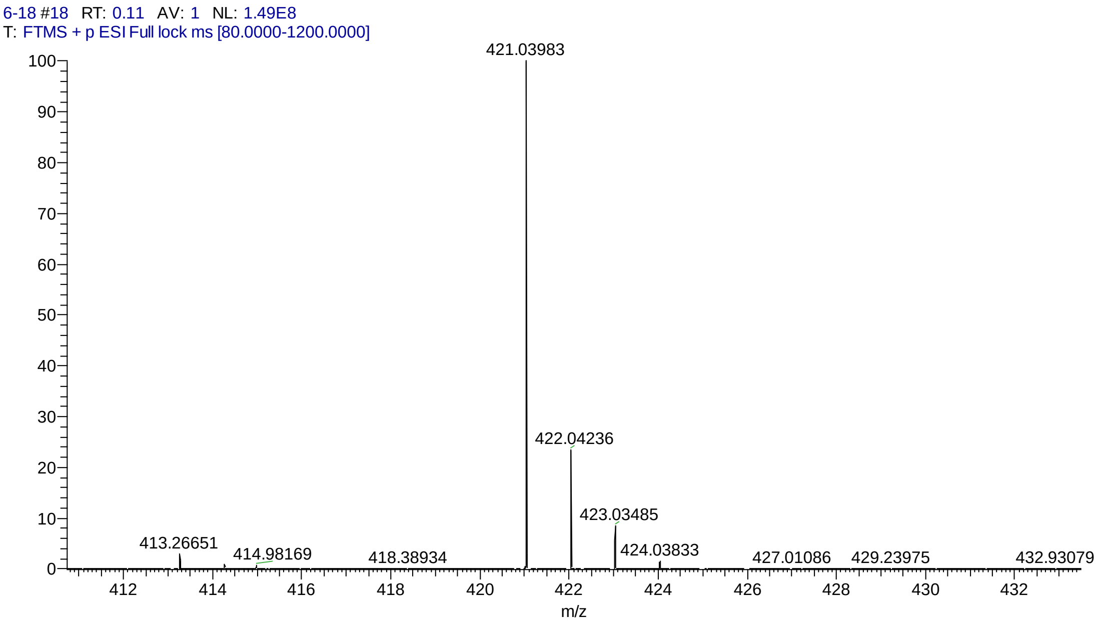

HRMS spectra of target compound **5s**

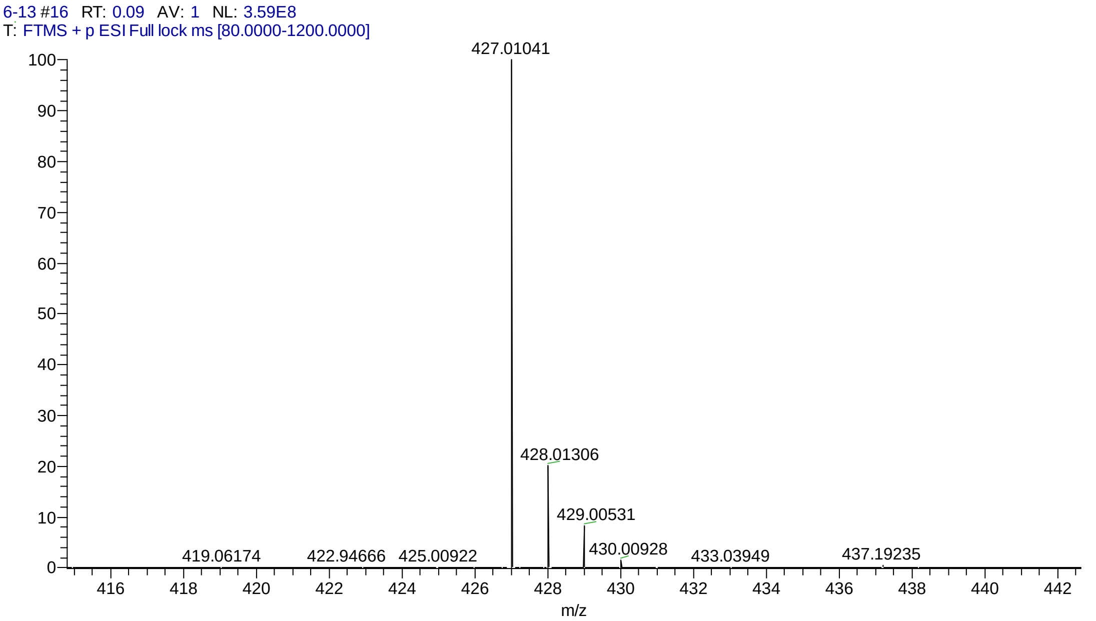

HRMS spectra of target compound **5t**

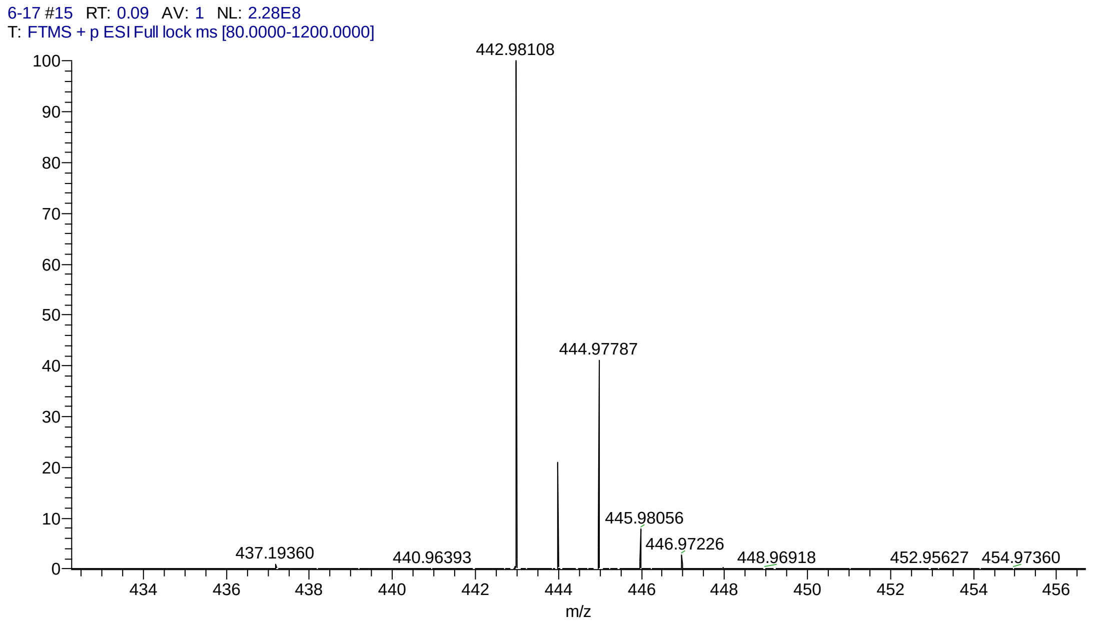

HRMS spectra of target compound **5u**

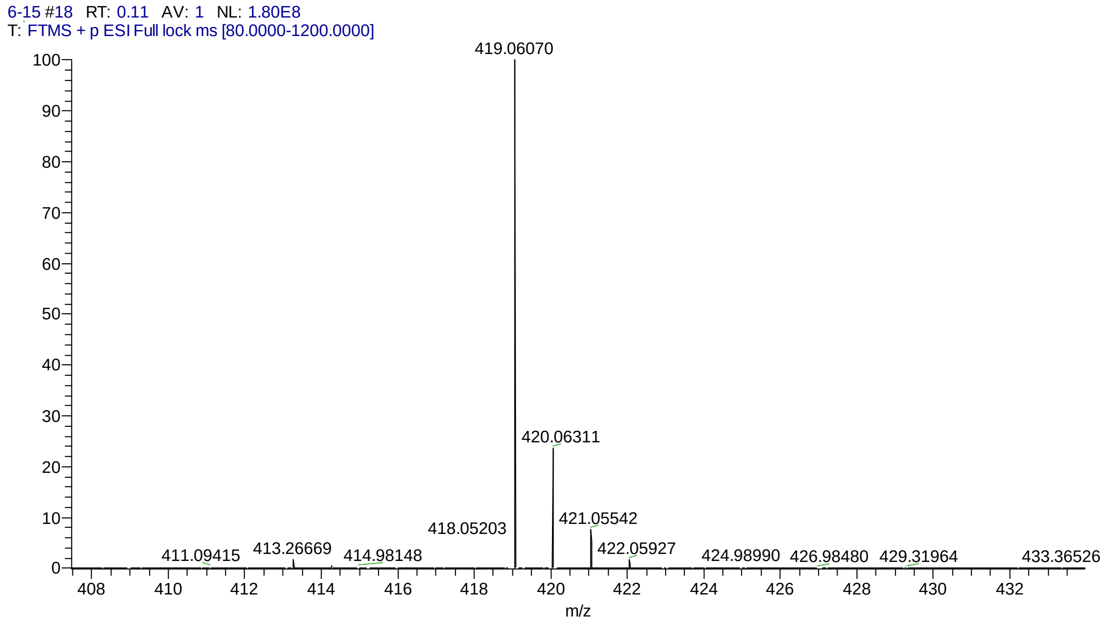

HRMS spectra of target compound **5v**
